# Supplementary material for: Shelter in Smoleń III – A unique example of stratified Holocene clastic cave sediments in Central Europe, a lithostratigraphic stratotype and a record of regional paleoecology
Source: PLoS One. 2020 Feb 6;15(2):e0228546. doi: 10.1371/journal.pone.0228546 (PMC7004378; doi:10.1371/journal.pone.0228546)
Supplement: Attachment — Submitted filename: PONE-D-19-27803_reviewer_NL_fin.pdf [file pone.0228546.s006.pdf]

Shelter in Smoleń III – a unique example of stratified Holocene clastic cave sediments in Central Europe, a lithostratigraphic stratotype and a record of regional paleoecology

--Manuscript Draft--

**Manuscript Number:**	PONE-D-19-27803
**Article Type:**	Research Article
**Full Title:**	Shelter in Smoleń III – a unique example of stratified Holocene clastic cave sediments in Central Europe, a lithostratigraphic stratotype and a record of regional paleoecology
**Short Title:**	Holocene clastic cave sediments of Shelter in Smoleń III (Poland)
**Corresponding Author:**	Maciej T. Krajcarz, Ph.D.<br>Instytut Nauk Geologicznych Polskiej Akademii Nauk<br>Warszawa, POLAND
**Keywords:**	Quaternary; Holocene; Pleistocene; sedimentology; geology; stratigraphy; lithostratigraphy; radiocarbon dating; age model; speleology; cave; rockshelter; shelter; cave sediments; cave deposits; fossil fauna; mollusks; rodents; chiropterans; paleoecology; paleoenvironment; paleogeography; bioturbation; archaeology; burial; human occupation; Central Europe; Poland; Polish Jura; Kraków-Częstochowa Upland
**Abstract:**	A cave site Shelter in Smoleń III (southern Poland) contains an approximately 2-m-thick stratified sequence of Upper Pleistocene and Holocene clastic sediments, unique for Central Europe. The sequence contents abundant fossil fauna, including mollusk, rodent and bat remains. The cave sites with long profiles of subfossil fauna present a great value for reconstructions of regional terrestrial paleoenvironment. We explore the stratigraphy of this site through analyses of the lithology and geochemistry of sediments, radiocarbon dating of faunal and human remains and charcoals, and archaeological study, as well as the paleoecology derived from the taxonomic composition of fossil faunal assemblages. Our data show that the entire period of the Holocene is recorded in the rockshelter, which makes that site an exceptional and highly valuable case. We present paleoenvironmental reconstructions of regional importance, and we propose to regard Shelter in Smoleń III as a regional stratigraphic stratotype of Holocene clastic cave sediments.
**Order of Authors:**	Maciej T. Krajcarz, Ph.D.
	Marcin Szymanek
	Magdalena Krajcarz
	Andrea Pereswiet-Soltan
	Witold P. Alexandrowicz
	Magdalena Sudoł-Procyk
**Additional Information:**	
**Question**	**Response**
**Financial Disclosure**<br><br>Enter a financial disclosure statement that describes the sources of funding for the work included in this submission. Review the submission guidelines for detailed requirements. View published research articles from *PLOS ONE* for specific examples.<br><br>This statement is required for submission	The funders had no role in study design, data collection and analysis, decision to publish, or preparation of the manuscript.

Powered by Editorial Manager® and ProduXion Manager® from Aries Systems Corporation

and **will appear in the published article** if the submission is accepted. Please make sure it is accurate.<br><br>**Unfunded studies**<br>Enter: *The author(s) received no specific funding for this work.*<br><br>**Funded studies**<br>Enter a statement with the following details:<br>• Initials of the authors who received each award<br>• Grant numbers awarded to each author<br>• The full name of each funder<br>• URL of each funder website<br>• Did the sponsors or funders play any role in the study design, data collection and analysis, decision to publish, or preparation of the manuscript?<br>• **NO** - Include this sentence at the end of your statement: *The funders had no role in study design, data collection and analysis, decision to publish, or preparation of the manuscript.*<br>• **YES** - Specify the role(s) played.<br><br>* typeset	
**Competing Interests**<br><br>Use the instructions below to enter a competing interest statement for this submission. On behalf of all authors, disclose any competing interests that could be perceived to bias this work—acknowledging all financial support and any other relevant financial or non-financial competing interests.<br><br>This statement **will appear in the published article** if the submission is accepted. Please make sure it is accurate. View published research articles from *PLOS ONE* for specific examples.	The authors have declared that no competing interests exist.

**NO authors have competing interests**<br><br>Enter: *The authors have declared that no competing interests exist.*<br><br>**Authors with competing interests**<br><br>Enter competing interest details beginning with this statement:<br><br>*I have read the journal's policy and the authors of this manuscript have the following competing interests: [insert competing interests here]*<br><br>* typeset	
**Ethics Statement**<br><br>Enter an ethics statement for this submission. This statement is required if the study involved:<br><br>• Human participants<br>• Human specimens or tissue<br>• Vertebrate animals or cephalopods<br>• Vertebrate embryos or tissues<br>• Field research<br><br>Write "N/A" if the submission does not require an ethics statement.<br><br>General guidance is provided below. Consult the submission guidelines for detailed instructions. **Make sure that all information entered here is included in the Methods section of the manuscript.**	Field research - permission for archaeological excavation granted by the Regional Heritage Office (Polish: Wojewódzki Urząd Ochrony Zabytków w Katowicach) (document No: K-AR.5161.61.2012.JP, ID: 3110, permission No 74/2012), and by the Polish National Forests, regional office in Olkusz (Polish: Lasy Państwowe - Nadleśnictwo Olkusz) as the land owner and forest manager (permission No: ZG5/503-1/2012 and ZG5/503-1/2013).

Powered by Editorial Manager® and ProduXion Manager® from Aries Systems Corporation

**Format for specific study types**<br><br>**Human Subject Research (involving human participants and/or tissue)**<br>• Give the name of the institutional review board or ethics committee that approved the study<br>• Include the approval number and/or a statement indicating approval of this research<br>• Indicate the form of consent obtained (written/oral) or the reason that consent was not obtained (e.g. the data were analyzed anonymously)<br><br>**Animal Research (involving vertebrate animals, embryos or tissues)**<br>• Provide the name of the Institutional Animal Care and Use Committee (IACUC) or other relevant ethics board that reviewed the study protocol, and indicate whether they approved this research or granted a formal waiver of ethical approval<br>• Include an approval number if one was obtained<br>• If the study involved *non-human primates*, add *additional details* about animal welfare and steps taken to ameliorate suffering<br>• If anesthesia, euthanasia, or any kind of animal sacrifice is part of the study, include briefly which substances and/or methods were applied<br><br>**Field Research**<br><br>Include the following details if this study involves the collection of plant, animal, or other materials from a natural setting:<br>• Field permit number<br>• Name of the institution or relevant body that granted permission	
**Data Availability**<br><br>Authors are required to make all data underlying the findings described fully available, without restriction, and from the time of publication. PLOS allows rare exceptions to address legal and ethical concerns. See the PLOS Data Policy and FAQ for detailed information.	Yes - all data are fully available without restriction

A Data Availability Statement describing where the data can be found is required at submission. Your answers to this question constitute the Data Availability Statement and **will be published in the article**, if accepted.<br><br>**Important:** Stating 'data available on request from the author' is not sufficient. If your data are only available upon request, select 'No' for the first question and explain your exceptional situation in the text box.<br><br>Do the authors confirm that all data underlying the findings described in their manuscript are fully available without restriction?	
**Describe where the data may be found in full sentences. If you are copying our sample text, replace any instances of XXX with the appropriate details.**<br><br>• If the data are **held or will be held in a public repository**, include URLs, accession numbers or DOIs. If this information will only be available after acceptance, indicate this by ticking the box below. For example: *All XXX files are available from the XXX database (accession number(s) XXX, XXX.*).<br>• If the data are all contained **within the manuscript and/or Supporting Information files**, enter the following: *All relevant data are within the manuscript and its Supporting Information files.*<br>• If neither of these applies but you are able to provide **details of access elsewhere**, with or without limitations, please do so. For example:<br><br>*Data cannot be shared publicly because of [XXX]. Data are available from the XXX Institutional Data Access / Ethics Committee (contact via XXX) for researchers who meet the criteria for access to confidential data.*<br><br>*The data underlying the results presented in the study are available from (include the name of the third party*	All relevant data are within the manuscript and its Supporting Information files

and contact information or URL).<br>• This text is appropriate if the data are owned by a third party and authors do not have permission to share the data.<br><br>* typeset	
Additional data availability information:	Tick here if the URLs/accession numbers/DOIs will be available only after acceptance of the manuscript for publication so that we can ensure their inclusion before publication.

Powered by Editorial Manager® and ProduXion Manager® from Aries Systems Corporation

Manuscript
Click here to download Manuscript ShSIII final main text 2019-10-03.doc

Shelter in Smoleń III – a unique example of stratified Holocene clastic cave sediments in Central Europe, a lithostratigraphic stratotype and a record of regional paleoecology

Maciej T. Krajcarz [1*], Marcin Szymanek [2], Magdalena Krajcarz [3], Andrea Pereswiet-Soltan [4], Witold P. Alexandrowicz [5], Magdalena Sudoł-Procyk [3]

[1] Institute of Geological Sciences, Polish Academy of Sciences. Twarda 51/55, 00-818 Warszawa (Poland), mkrajcarz@twarda.pan.pl

[2] University of Warsaw, Faculty of Geology. Żwirki i Wigury 93, 02-089 Warszawa (Poland), m.szymanek@uw.edu.pl

[3] Institute of Archaeology, Nicolaus Copernicus University in Toruń. Szosa Bydgoska 44/48, 87-100 Toruń (Poland), magkrajcarz@umk.pl, sudol@umk.pl

[4] Department of Paleozoology, Institute of Evolutionary Biology and Ecology, Faculty of Biological Sciences, University of Wrocław. Sienkiewicza 21, 50-335 Wrocław, (Poland), pereswiet_soltan@yahoo.it

[5] AGH University of Science and Technology, Faculty of Geology, Geophysics and Environment Protection, Chair of General Geology and Geotourism. Al. Mickiewicza 30, 30-059 Kraków (Poland), wpalex@geol.agh.edu.pl

* corresponding author

Abstract

A cave site Shelter in Smoleń III (southern Poland) contains an approximately 2-m-thick stratified sequence of Upper Pleistocene and Holocene clastic sediments, unique for Central Europe. The sequence contents abundant fossil fauna, including mollusk, rodent and bat remains. The cave sites with long profiles of subfossil fauna present a great value for reconstructions of regional terrestrial paleoenvironment. We explore the stratigraphy of this site through analyses of the lithology and geochemistry of sediments, radiocarbon dating of faunal and human remains and charcoals, and archaeological study, as well as the paleoecology derived from the taxonomic composition of fossil faunal assemblages. Our data show that the entire period of the Holocene is recorded in the rockshelter, which makes that site an exceptional and highly valuable case. We present paleoenvironmental reconstructions of regional importance, and we propose to regard Shelter in Smoleń III as a regional stratigraphic stratotype of Holocene clastic cave sediments.

Introduction

Cave sediments are important source of information for understanding the history of terrestrial geoecosystems. Due to their concave morphology and solid walls, caves serve as sediment traps with the ability to continuously collect sediments for millennia. Preserved sequences record long time intervals and are able to survive destructive geomorphological events, such as ice sheet advance, floods or deflation. Although the stratigraphic resolution of cave sediments is usually much lower than in subaqueous deposits, such as lacustrine or peat bog sediments, caves are among the rare landforms

that record subaerial terrestrial conditions. In particular, data on past terrestrial fauna are well protected in caves. This outcome is not only because caves serve as shelters for animals and are natural places of deposition of animal remains but also because a calcareous environment favors preservation of fossil bones and shells.

Caves have also been used by humans in prehistoric, historic and recent times as living places, temporary shelters, storerooms or sacral objects[1,2]. Therefore, they are important archaeological sites, often with multiple cultural levels preserved in superposition and delivering data on the local or regional development of human beings and their culture. For this reason, it is crucial not only to recognize the stratigraphy of cave sediments in particular sites but also to detect regional lithological variability and similarities between sites. This would help to formulate more general stratigraphic schemes that can be used for further inter- and intraregional correlations.

Kraków-Częstochowa Upland in southern Poland is a karstic region with a very large number of caves and rockshelters[3]. Most of these features contain clastic sediments, in some cases studied by archaeologists, geologists and paleontologists. The lithology and stratigraphy of Pleistocene sediments were recognized in a number of sites in the region[4–12]. In opposition to the Pleistocene series, the Holocene sediments were poorly studied. The main reason is the focus of the scientific community on the Pleistocene and Paleolithic issues. An additional reason is that the internal stratification of the Holocene series in caves of the region is usually poor or completely lacking, and at the most sites, the entire Holocene sequence is limited to only one stratum[4–7,12–15]. This stratum has usually been described as a dark loam or humus layer. Explanation of this faint stratification is beyond the scope of this paper; however, the most likely reason is past human activity, which was especially intensive during the Medieval and post-Medieval periods[1,2,12]. To date, only a few sites with complex Holocene stratigraphy have been

discovered in Kraków-Częstochowa Upland: Żytnia Skała Rockshelter[16]; Nad Mosurem Starym Duża Cave[17]; the cave above the Słupska Gate[18]; Żarska Cave, which was excavated recently[19]; most likely Zegar Cave[20]; Perspektywiczna Cave, which is still under excavation[11,21]; and Shelter in Smoleń III, which is the subject of this paper. The last site bears a complex stratified series of Late Glacial and Holocene cave sediments, which was found during an archaeological excavation from 2012-2013. Rich fossil material of snails, rodents and bats allows reconstructing the paleoecological and paleoclimatic conditions and using them as the proxies to build a comprehensive litho-bioclimato-stratigraphic scheme for this rockshelter.

Localization

Shelter in Smoleń III (ShSIII) is situated near Smoleń village (Pilica comm., Zawiercie dist., southern Poland), in the central part of Kraków-Częstochowa Upland (Fig 1), in the microregion called Ryczów Upland[22]. The rockshelter is situated on the right side of the currently dry Wodąca Valley, 445 m a.s.l., E 19°40′27″, N 50°26′02″. Two entrances are situated next to one another; they are exposed to the NE (Fig 2) and open to a small plateau in front of the rockshelter. The rockshelter is most likely a remnant of a larger collapsed cave system. Its total length is 8.8 m, including the branches[23], and the surface of sedimentary fill is approximately 11 m^2. The entire interior is controlled by external weather and accessible to daylight, except for the rearmost area.

The rockshelter was named *Schronisko w Smoleniu III* (Shelter in Smoleń III) and initially listed under the inventory number 392[24], then IV.C.12[3], and finally J.Cz.IV-

04.44[23]. The last number and the name are listed in the governmental database of the caves of Poland (http://jaskiniepolski.pgi.gov.pl/).

Fig 1. Localization of ShSIII in Central Europe (left map), within Kraków-Częstochowa Upland (central map) and at the digital terrain model of the nearby area (right map). Legend: a – ShSIII; b – other important cave sites of Holocene sediments and fossil fauna in the region (CSG – Cave above the Słupska Gate, PC – Perspektywiczna Cave, MSDC – Nad Mosurem Starym Duża Cave, ŻC – Żarska Cave, ŻSR – Żytnia Skała Rockshelter); c – other caves and rockshelters (after the Polish governmental database http://www.jaskiniepolski.pgi.gov.pl/); d – microregion of Kraków-Częstochowa Upland (according to Kondracki[22]); e – the largest cities; f – boundary of Natura 2000 protected area *Ostoja Środkowojurajska*; g – Smoleń Natural Reserve.

Fig 2. The entrances of ShSIII. A view from north-east, from the small plateau in front of the rockshelter. Two entrances are indicated with the arrows. Photo made in April 2014 by MS-P.

Regional geology

The region is characterized by a variable topography with steep high limestone tors and cliffs and deeply cut valleys. The core of the microregion are the Upper Jurassic massive limestones, partially covered by red desert sands and loams dated to Paleogene/Neogene[25] and sandy deposits attributed to the fluvio-periglacial facies of the Middle Pleistocene[26,27]. Near the site, the sands are covered by loess deposits, which

become thicker and tend to form a continuous coverage toward the east[28]. The karstic cavities (caves and rockshelters) in the microregion are filled with the Upper Pleistocene and Holocene sediments[7,20,29,30]. The most complex stratigraphic sequence has been found in the nearby Biśnik Cave, situated 1.1 km to southwest, where pre-Pleistocene, Middle-to-Upper Pleistocene and Holocene sediments have been preserved[10,31]. However, the Holocene sediments of Biśnik Cave exhibit reduced thickness and weak lithological variability.

Archaeological site

The rockshelter was recognized as an archaeological site in 2012, during the test pitting ~~realized as a part of~~ the research project focused on the ancient settlement pattern in the region[32]. The excavation continued in 2013 and revealed a multi-episode sequence of human activity[33–35]. It is noteworthy that only minor attention has been given to the Holocene archaeological sites in caves of Kraków-Częstochowa Upland until last years, especially to the stratigraphic situation of the archaeological finds. Therefore, the thorough investigation of ShSIII, including the modern excavation standards, makes this site an important record of the Holocene human activity in the region.

Material and methods

Excavation and sampling

The site was excavated from 2012-2013 according to the archaeological standards of the test pitting. Excavation covered 10 squares of the archaeological grid (approximately 7 m^2 of surface in total, Fig 3) and reached the bedrock, revealing a

maximum 2.6-m-thick sequence of clastic sediments[32]. The deposits were divided into archaeological layers according to differences in the color, consistency and amount of limestone debris observed in the field. These layers well reflect the geological stratification, and in some cases, they represent soil horizons. The excavation was performed by removing 10-cm-thick intervals of sediment (or 5-cm-thick where the concentration of artifacts or fossils was high) from the squares. The arrangement of layers was recorded by drawing and photographing for each interval. The following finds and samples were collected:

- Archaeological artifacts – each find was collected and its position recorded in 3D standard.
- Macrofossils – each bone, shell and charcoal detected at the site was collected and its position recorded in 3D standard.
- Paleontological samples – approximately 5-liter samples of the fine fraction of sediments (macrofossils collected separately, larger clasts removed by hands) were collected from each square, separately for each layer from each 5- or 10-cm interval. The samples were then wet-sieved through a 0.5-mm mesh, and any paleontological material was picked by hand.
- Geological samples – 36 samples of sediment with undisturbed grain size composition (i.e., fine fraction collected together with associating pebbles, cobbles and boulders), approximately 7 liters each, were taken from the walls of archaeological trenches according to methodology by Madeyska-Niklewska[36]. At least one sample was taken from each layer if possible, and in the case of layers with greater thickness, several samples were taken from one layer.

- Luminescence dating samples – four 0.5-liter samples of fine fraction were taken under dark conditions from selected layers with low amounts of limestone clasts and secured in opaque containers.

The collections of archaeological material and vertebrate remains are stored at the Institute of Archaeology, Nicolaus Copernicus University in Toruń (Poland). The collection of mollusk remains is stored in the Faculty of Geology, University of Warsaw (Poland).

Fig 3. Plan of ShSIII. The cross-sections A’-B’, B’-C’ and D’-E’ are shown in Fig 4.

Lithological and geochemical analyses

Geological samples were dried and separated into fractions with a set of sieves (2, 4, 10, 20, 40 and 80 mm meshes). Macroscopic archaeological or paleontological material was removed by hand. The coarse fraction (>2 mm), composed almost exclusively of limestone clasts, was soaked in sodium phosphate solution for 24 h to dissolve clay aggregates and then washed with a shower on a 2-mm sieve. After drying, the material was weighted to calculate the amount of each fraction. The fine fraction (<2 mm) was homogenized by mixing and divided into smaller samples by quartering. Approximately 5 g samples of fine fractions were analyzed on a Malvern Mastersizer 2000 laser diffractometer with a HydroG adapter (analysis subcontracted to the Department of Geoecology and Palaeogeography, Maria Curie-Skłodowska University in Lublin). The procedure followed the widely accepted methodology[37].

Morphological classification of limestone clasts was performed on the >20 mm fraction, according to published methodology[36,38]. Four morphological classes were

distinguished: A – sharp-edged (or angular) clasts; Ba – slightly smoothed (subangular) clasts with blunt edges; Bb – smoothed clasts with rounded edges (no edges recognizable), but with still-identifiable flat facets; C – highly smoothed (rounded-like) clasts with no recognizable edges or flat facets.

The chemical composition of the sediments was determined using approximately 1-g samples of homogenized <2 mm fraction. The analytical method was inductively coupled plasma mass spectrometry (ICP-MS) performed in the Bureau Veritas Minerals Laboratory (Vancouver, Canada) according to the published procedure[39]. The content of organic matter was determined using another approximately 1-g aliquot of <2 mm fraction via oxygenation in 30% H_2O_2 for 24 h on a shaking plate[40]. If the reaction was still ongoing (bubbling or dark color), an additional portion of H_2O_2 was added for the next 24 h. The content of organic matter was calculated as the difference between the weight of the dry sample and that of the dry residue after the reaction.

Chronometric dating

Radiocarbon dating

AMS radiocarbon dating was conducted in the Poznań Radiocarbon Laboratory (Poland). The dating material included snail shells, human and animal bones, charcoals and one example of organic char residue preserved on the internal surface of the ceramic pot[33]. In the case of snails, each dating was performed on one identifiable shell of *Isognomostoma isognomostomos*. Shells of *Discus ruderatus* were also dated, but due to their small size, several shells from the same layer, square meter and depth were mixed together. A small aliquot of each shell sample was checked with X-ray diffraction (at the Faculty of Geology, University of Warsaw, Poland) prior to dating to exclude any material with negative effect of aragonite-to-calcite recrystallization. Only

samples evidencing pure aragonite were dated. Chemical pretreatment followed Brock et al.[41]. In the case of bones, the dated fraction was collagen extracted according to Goslar et al.[42]. Small aliquots of extracted collagen were analyzed with a CHNS elemental analyzer, and atomic C:N ratios was calculated to check the collagen quality. The range of 2.9-3.6 was assumed acceptable according to the literature[43,44]. For three samples, the collagen yield was too low for both elemental analysis and radiocarbon dating, and these samples were dated without the C:N ratio being confirmed. In the case of charcoals, cellulose was extracted according to AAA method[41].

All dates were calibrated using the OxCal v. 4.3 software package[45] versus the IntCal'13 radiocarbon calibration curve[46]. Calibrated dates are presented as ky BP (i.e., thousand years before 1950), as the ranges within a 94.5% probability interval. The OxCal v. 4.3 sequence model was applied to estimate the chronology of layer boundaries. It followed the procedure used by Krajcarz et al.[47], based on the widely accepted methodology[48,49].

Luminescence dating

The luminescence dating of samples was subcontracted to the Department of Geoecology and Palaeogeography, Maria Curie-Skłodowska University (Lublin). Post-IR $IRSL_{290}$ dating was performed on the polymineral fine grains (4-11 μm). The extraction procedure followed the accepted methodology[50]. Equivalent doses were determined using a post-IR IRSL protocol[51].

Paleozoological analyses

Mollusks, rodents and bats are the most numerous groups of fossil animals in ShSIII and therefore were chosen for the paleozoological analysis to provide supportive paleoecological and paleoclimatic data for stratigraphy. In addition to these groups, the

fossil material also comprises the remains of amphibians, reptiles, birds, insectivores and large mammals including humans. For the purpose of paleoecological analysis, layer 3 has been divided into two parts (3 lower and 3 upper) due to its great thickness and abundant rodent and mollusk remains.

Identification of mollusk remains

Standard methods[52–55] were applied for mollusk analysis. The shells collected during sieving were carefully cleaned. All completely preserved shells and their identifiable fragments were identified under a binocular microscope using taxonomical keys[56–58] and counted applying the schemes for broken individuals[52,55]. The number of individuals was counted separately for each layer. For some damaged individuals, only the genus or family levels were determined, and the calcareous plates of slugs were counted together under the heading of Limacidae.

Identification of rodent remains

The rodent fossil assemblage consists of single bones and isolated teeth. Specimens were identified in terms of skeletal elements and species when possible. The taxonomical attribution of remains was based on the following diagnostic elements: first lower molars (Arvicolinae); mandibles, maxillae and isolated teeth (Muridae, Gliridae and Dipodidae); mandibles, maxillae, isolated teeth and diagnostic postcranial bones (*Cricetus cricetus, Sciurus vulgaris*). Remains were identified under a binocular microscope with use of the comparative collection of recent rodents and following the general criteria given in the identification keys[59,60]. The separation of *Microtus arvalis* and *M. agrestis* was based on the method given by Nadachowski[61,62]. The taxonomic classification followed Wilson and Reeder[63] for all taxa except for *Clethrionomys*

glareolus (former *Myodes glareolus*) and *Lasiopodomys gregalis* (former *Microtus gregalis*), which were classified after later publications[64,65].

For each species, the number of identified specimens (NISP) and minimum number of individuals (MNI) were counted according to accepted method[66,67]. The MNI was counted separately for each layer.

Identification of bat remains

Specimens were identified, when possible up to the species level, on the basis of diagnostic traits of the hemimandible and/or the skull. In particular, the attribution of the taxonomic group was based on several different characteristics: the number of teeth, the shape of the hemimandible, the traits of the fourth lower and upper premolars and those of the upper and lower molars. Remains were identified and measured through a binocular microscope, comparing them with specimens belonging to a collection of recent bats. The general criteria given in the identification keys[68–70] were fulfilled, and the remains were classified taxonomically according to Wilson and Reeder[63]. The taphonomic parameters NISP and MNI were calculated according to the same method as for rodents.

Paleoecological reconstruction

Paleoecological inference was based on the environmental preferences of all mollusk, rodent and bat species found in the sediments. The structure of taphocoenoses and succession were presented on frequency bar diagrams. The diagrams were composed of both the absolute numbers of shells (or the MNI in the case of rodents) for samples containing less than 50 specimens and the percentages of the total sum for those with more than 50 specimens. In the case of bats, only the MNI values were shown due to low number of remains. Mollusk, rodent and bat assemblages were

distinguished on the basis of their structure and composition, supported by statistical clustering analyses. The clustering was done for samples (layers) with more than 10 shells or with MNI greater than 10 by applying a paired group algorithm and Horn's overlap index for abundance data using the PAST software package, version 3.22[71,72]. Both the character and succession of the assemblages were used in the paleoecological and paleoclimatic reconstructions.

To reconstruct the changes in ecological diversity in the stratigraphic sequence, the assemblages were analyzed via application of the richness and the Simpson index of evenness. In the case of rodents, specimens identified as *M. agrestis*/*arvalis* were excluded, while *Apodemus sylvaticus*/*flavicollis* and *Apodemus* sp. were counted as one taxon (genus *Apodemus*). The number of species per sample (layer) is a measure of richness. The greater the number of species present in a sample, the 'richer' the sample. The Simpson 1-D index was calculated as $1 - \Sigma(n_i/n)^2$, where n_i is the MNI of the particular taxon in an 'i-sample' (a layer) and n is the total number of MNIs in a sample (a layer)[72]. The index was calculated using the PAST software package, version 3.22. The index represents the probability that two individuals randomly selected from a sample will belong to different taxa.

To present the changes in the environment or landscape around the site, we used the environmental preferences of the species. Each mollusk taxon was assigned to one of four ecological groups[55]: F – shade-loving species; O – open-country species; M – mesophilous species; H – hygrophilous species. In the case of rodents, which usually inhabit a range of environments, we used the method of habitat weightings[73]. Each taxon was assigned to the habitat(s) where it can be found at present, with percentage weight(s) attributed for each of its habitat(s). The rodent fauna of ShSIII was assigned to eight habitat types based on a habitat identification mode provided in the literature[74–

[76], with modifications for habitats typical for Central and Northern Europe. The used habitats were as follows: Tu – tundra; OA – open anthropogenic cultivated areas; OD – open dry (steppes); OH – open humid (meadows); OW – open woodland (shrubs, forest margins, steppe-forests); FT – temperate forest (broadleaf forests, mixed forests); FB – boreal forests (taiga); Wa – water bodies. Data on the species distribution and their preferred habitats were taken from Mitchell-Jones et al.[77] and the International Union for Conservation of Nature (IUCN) Red List maps[78,79,88–96,80–87].

Bats with their flight ability are more mobile animals and this restricts their usefulness as the indicators of local paleoenvironment. However, some species which are sedentary and use old trees as summer roosts (i.e., *Barbastella barbastellus*, *Myotis bechsteinii* and *Plecotus auritus*) clearly indicate the presence of old-growth forests in the vicinity[97–99]. Bats hunting near water (*M. dasycneme* and *M. daubentonii*) indicate the presence of open water bodies[100,101].

Results

Lithostratigraphic sequence

The lithostratigraphic sequence comprises fourteen layers, some of them being lateral variants. A characteristic feature of ShSIII is the lithological dissimilarity of sediments between different parts of the site, despite its small size (Figs 4 and 5). Three lithostratigraphic zones could be distinguished on the basis of these dissimilarities: entrance, central and rear. They were recognized at the squares of archaeological grid as follows: entrance zone – C/4, D/4, E/4, and partially C/5; central zone – the remaining area of C/5 and entire D/5, E/5, D/6, E/6, F/6 and F/7; rear zone – C/8 and C/9 (see also Fig 3). Detail lithological and geochemical parameters are provided in Appendix S1.

Fig 4. The representative geological cross-sections through the sediments of ShSIII. Natural colors of moist sediments are shown (Munsell colors are provided in text). Vertical scale is in centimeters below the site datum, which is at the elevation of around 447 m a.s.l. Location of cross-sections is shown in Fig 3.

Figure 5. Basic lithological and geochemical parameters of the sediments of ShSIII. The data have been arranged by zones, layers and order of samples. For details see Appendix S1.

Entrance zone

The lower parts of the sedimentary sequence are similar at the entrance and central zones (Fig 4). The sediments of the upper part in the entrance zone are darker and more enriched in humus, as a result of the greater influence of external conditions and pedogenic processes. The total thickness of the sequence reaches 260 cm.

Layer 9 is a limestone debris composed of coarse sharp-edged clasts. The matrix is a yellowish brown to brown (Munsell color: dry 10YR 5/4-6/4, moist 10YR 5/3-5/4) silty loam to sandy silt. The limestone bedrock below the layer is cracked into angular blocks, and this regolith passes into layer 9 gradually, which is marked by decreasing size of blocks and occurrence of matrix. The Ca content is very high in the fine fraction, particularly in the lower part of the layer, where it reaches greater than 30% (Fig 5). This strongly suggests that the fine fraction is mostly composed of physically disintegrated limestone.

Layer 7 is a pale brown to brown (dry 10YR 6/3, moist 10YR 4/3) sandy silt. It is a structureless loess-like sediment with angular limestone clasts. Layer 9 passes indistinctly into layer 7, and the transition between the layers is marked by a gradual color change and decreasing amount of limestone clasts. The content of Ca in the fine fraction is very low (Fig 5), which indicates the allogenic source of material. At squares C/5 and partially D/5, a lens of dark silt (called layer 8; see the section "Anthropogenic layers") occurs in the upper part of layer 7. This intercalation enables dividing the layer locally into sublayers 7a (above layer 8) and 7b (below layer 8).

Layer 8 is a grayish brown to dark grayish brown (dry 2.5Y 5/2, moist 10YR 4/2) sandy silt. The texture is similar to that of layer 7, except for the dark color and the presence of charcoals inside layer 8.

Layer 3a is a grayish brown to black (dry 10YR 5/2, moist 10YR 2/1) silt. The amount of limestone clasts is greater than in layer 7. Angular blocks predominate, but smoothed clasts also occur. The concentration of P increases upward starting from layer 3a (Fig 5), indicating the increasing impact of zoogenic activity on the accumulation. The lower boundary is blurred, and the layer passes downward gradually into layer 7. Close to the walls of the rockshelter, the layer exhibits greater thickness, and its bottom is situated at lower elevation.

Layer 3 is a dark brown to very dark grayish brown (dry 10YR 3/2-4/3, moist 10YR 2/2-3/3) sandy silt. The amount and size of limestone clasts is lower here, and smoothed clasts occur in greater number (Fig 5). However, outside of the dripline (square meters C/4 and partially D/4), the large limestone blocks occur in the layer. Their surfaces and edges are smoothed. Indistinct trough cross-stratification is marked in the bottom part at square E/4.

Layer 1 is a very dark gray to black (dry 10YR 3/1, moist 2.5Y 2/1) silty sand. Limestone debris becomes less abundant and finer upward, and angular clasts are almost lacking, while smoothed, rounded-like clasts are relatively abundant. This layer exhibits unusually high concentrations of Pb and other toxic metals (Zn, Cd, Ag, Sb, Bi, Sn, see Fig 5), suggesting an increased impact of human activity. Another characteristic of the layer is a high sand fraction.

Central zone

The lithological variability is the greatest in this area, and the stratigraphy is the most complex, with up to eight distinct strata preserved in superposition (Fig 4). The lower part of the sequence shares lithological characteristics with the entrance zone, but in the upper part, the sediments are different. The sedimentary fill achieves a thickness similar to that of the sediments in the entrance zone (230 cm at maximum).

The sequence starts with a limestone debris containing very pale brown to yellowish brown (dry 10YR 8/3, moist 10YR 5/6) silty to sandy matrix (called **layer 9**), which is a continuation of layer 9 from the entrance zone. The clasts are finer here, but angular shapes still predominate (Fig 5). The layer reaches the greatest thickness (110 cm) at squares E/6, F/6 and F/7, where it fills the narrow and deep depression of the bedrock, probably a remnant of a vadose canyon.

The overlying sediment (called **layer 7**) is a continuation of layer 7 from the entrance zone. It is a yellowish brown to pale brown and brown (dry 10YR 6/3, moist 10YR 4/3 – 5/4) loess-like sandy silt with limestone clasts. The clasts are mostly angular and become less abundant and finer upward (Fig 5). The concentration of Ca in the fine fraction is very low (Fig 5), which suggests an allochthonous source of fine material. The lower boundary of this layer is indistinct, and layer 9 passes gradually into layer 7.

Similar to the entrance zone, locally (at square D/5, where layer 8 occurs), the layer can be divided into sublayers 7a (above layer 8) and 7b (below). Sublayer 7a has a lighter, more yellowish color (dry 10YR 6/3, moist 10YR 5/4) and lower amount of limestone clasts compared with sublayer 7b (color: dry 10YR 6/3, moist 10YR 4/3). The lower boundary of sublayer 7a is not always readable, but locally, especially in contact with layer 8, it is sharp and erosional.

Layer 8 is probably a continuation of layer 8 from the entrance zone. It is a grayish brown to dark grayish brown (dry 2.5Y 5/2, moist 10YR 4/2) sandy silt. It occurs in restricted area, limited to square D/5. The layer seems to fill the shallow depressions in sublayer 7b. The remains of a hearth are preserved in the lower part of layer 8 and labeled as sublayer 8a.

Layer 6 is a light brownish gray to dark grayish brown (dry 10YR 6/2-6/3, moist 10YR 4/2 – 2.5Y 5/2) sandy silt. The amount, size and morphology of limestone clasts are similar to layer 7 (Fig 5). The concentrations of Ca and P increase from layer 6 upward, recording the increasing importance of autogenic deposition and zoogenic activity. The lower boundary of layer 6 is sharp and undulating. Internal sedimentary structures in a form of trough cross-stratification are weakly marked inside the layer.

Layer 5 is a grayish brown to dark grayish brown (dry 10YR 5/2-5/3, moist 10YR 4/2-4/3) silty loam to sandy silt. The amount of limestone debris is greater than in layer 6 (Fig 5). The clasts are smoother, and even rounded-like ones occur. The lower boundary is discordant, sharp and undulating. The trough cross-stratification is locally distinct, with bedding inclined toward the entrance.

Layer 5a is a whitish to grayish brown and dark grayish brown (dry 10YR 5/2-8/2, moist 10YR 4/3-6/3) structureless silty loam to clay silt. The sediment is clearly lighter

in color than those of the lower strata. Its lower boundary is sharp, and the layer lies discordantly on lower sediments. Locally, layers 5 and 6 are not preserved, and layer 5a lies directly on layer 7. The amount of limestone clasts is relatively low, and the clasts are fine and smoothed (Fig 5). The concentrations of Ca and P are among the highest in the entire sequence. The layer is not continuous and is lacking at square meters D/5, D/6 and E/6.

Layer 4 is a very pale brown to brown (dry 10YR 7/3, moist 10YR 5/3) structureless (massive) silty loam. The amount of limestone clasts is low; their morphology is similar to that in layer 5a (Fig 5). The clay content increases in the upper part of layer 5a and stays high in layer 4 (Fig 5). The concentration of Ca is similar to that in layer 5a, whereas the concentration of P is slightly lower (Fig 5). The lower boundary is indistinct and gradual.

Layer 2 is similar to layer 4 in terms of the grain size composition and limestone clast morphology (Fig 5) but differs in color, being more grayish (dry 2.5Y 7/2, moist 2.5Y 4/2). The concentrations of Ca and P are still relatively high but lower than in layer 4. The boundary between layers 2 and 4 is indistinct and gradual.

The sequence is topped with **layer 1a**, which is a lateral variant of layer 1 from the entrance zone. This sediment is more brownish than layer 1 (dry 2.5Y 3/2, moist 2.5Y 2/2). The thickness of layer 1-1a decreases toward the rockshelter interior, such that the maximum thickness of layer 1 is 60 cm and that of layer 1a is up to approximately 30 cm. Upper part of layer 1a cover the archaeological feature (layer A), and due to lithological similarities of these two units, the boundaries between them are difficult to follow.

Rear zone

The sequence in the rear zone has a reduced thickness, achieving 120 cm at maximum (Fig 4). Sediments of this zone were recognized in a limited area of approximately 1 m^2. The rockshelter is shaded, and sunlight does not reach this area. The corridor declines downward to the south and becomes narrower, down to the width not accessible for penetration by an adult human. The concentration of K is low in this zone, which indicates lower infiltration from the top strata, possibly as a result of low rainwater supply in the deeper part of the rockshelter.

The sequence starts with **layer 11**, which is a complex stratum of bright reddish-brown color. The average grain size composition indicates a silty loam texture, but the layer is composed of numerous diapirs of brown (dry 7.5YR 5/4, moist 7.5YR 5/4) clay and lenses of reddish brown to strong brown (dry 7.5YR 6/6, moist 7.5YR 5/6) silty sand. This sandy component is most likely an admixture from layer 10. Limestone clasts are absent. The sediment is strongly enriched in Fe and depleted in Ca (Fig 5), what indicates an allogenic origin. High concentrations of Fe and other geochemically related metals (Mn, Co, Zn, Pb, Cu, Mo, Th, Cd, Sb, and REE) are characteristic features of this sediment and are responsible for its reddish color. The thickness is greater than 30 cm.

Layer 10 is a yellow to brownish yellow (dry 10YR 7/5, moist 10YR 6/7) silty sand. Sandy grains are composed mostly of quartz. Such a mineral composition, together with the lack of limestone clasts and very low concentration of Ca (Fig 5), indicates an allogenic origin. The sediment is preserved only in pocket-like or tongue-like structures immersed in layer 11. The contact between the two layers, although not in a primary position, is sharp, what indicates the erosional boundary at the bottom of layer 10. The original thickness of the layer is difficult to establish due to disturbances, but it can be estimated to be greater than 5 cm.

Layer 9 is a 15-cm-thick horizon of coarse subangular limestone blocks, with yellowish brown to dark yellowish brown (dry 10YR 5/3, moist 10YR 4/4) silty loam filling the space between blocks. The lower boundary is sharp and erosional. The vertical orientation typical for layers 11 and 10 is not visible here. High concentrations of trace metals, similar to those exhibited by layer 11 (Fig 5), indicate that layer 11 served as important source of material. This layer most likely forms the lateral continuation of layer 9 from the entrance and central zones.

That layer passes upward gradually into **layer 4**. This is a very pale brown to yellowish brown (dry 10YR 7/3-7/4, moist 10YR 5/4-5/5) silt or clay silt, passing upward into silty loam. This layer is a lateral continuation of layer 4 from the central zone. The concentrations of Ca and P are high and increase toward the top of the layer.

Layers 2 and 1a are lateral continuations of layers 2 and 1a, respectively, from the entrance zone and share lithological characteristics with those strata.

Anthropogenic layers

Two anthropogenic strata were recognized between the natural strata and were labeled as sublayer 8a of layer 8 and layer A.

Sublayer 8a is a black (dry 10YR 4/1, moist 10YR 2/1) sandy silt. It occurs in the lower part of layer 8 and represents its darker variant. The sublayer has a form of a thin lens with a thickness of approximately 10 cm and lies at the bottom of a shallow depression dug into layer 8 and reaching sublayer 7b. The presence of charcoals indicates that this deposit is a remnant of a hearth.

Layer A is a sediment of intermediate characteristics between layers 1, 1a, 2 and 3. It is a dark brown to very dark grayish brown (dry 10YR 3/1-4/3, moist 10YR 2/2-3/2) silty sand. It forms a backfill of an anthropogenic feature. Its lower boundary is sharp

and distinct. Stratigraphically, the feature is situated inside layer 1-1a, as its walls cut layer 1 or 1a, and at some square meters, it is covered by the upper part of layer 1a, with an indistinct boundary between the layers. The bottom of the feature reaches layer 2 or 3 (at different square meters) and occasionally layers 4 and 5a.

Chronometric data

A total of 29 radiocarbon dates are accessible for the ShSIII (Table 1). The dates cover the interval from 14.1 to 0.6 ky BP, i.e., almost the entire Holocene and Late Glacial of the Last Glaciation (Fig 6). In case of bones, the atomic C:N ratio in extracted collagen is in the widely accepted 2.9-3.6 range[43,44] for all tested samples (Table 1; only two samples yielded too low amounts of collagen to check the C:N ratio), confirming that the analytical fraction was pure. Four TL dates were also obtained for layers 7b, 6 and 5a (Table 2).

To build the sequence model in OxCal, we used only the radiocarbon dates from the central zone, to achieve the data for a single stratigraphic column and avoid problems due to lateral correlations. Moreover, the central zone delivered most of the dates, while only two radiocarbon dates were obtained from material from the entrance zone. The archaeological feature (layer A) was excluded because its backfill includes redeposited material from the lower layers and may disturb the model of deposition. A date from layer 1a (Poz-53301) was also excluded, as this date was obtained from a human bone, which was most likely redeposited from the grave, i.e. from layer A. The model including all dates from the central zone did not run until the chronologically discontinuous dates in were removed. The dates in disorder were mostly those from layers 4, 5a and 5. There are several possibilities for selecting dates for removal. The least-invasive method is to remove three dates, Poz-61237, Poz-61305 and Poz-53303

(marked in Fig. 6 as "intrusive material?"), and this method was applied to build the model (Table 3).

The dates from layer A (backfill of archaeological feature) and from a human bone from layer 1a (Poz-53301) were used to build a separate sequence model to determine the boundaries of the phases of human activity. The dates were attributed in an arbitral manner to three phases of human activity (PHA): PHA 2 – Bronze Age; PHA 3 – pre-Roman to Roman Period; and PHA 4 – Late Middle Ages (PHA 1 was reserved for the Late Paleolithic phase). The model for PHA boundaries gave very good overall agreement index $A_{overall}$=96.4%. The model for the boundaries of the natural layers gave weaker agreement index, $A_{overall}$=78.8%, yet still in the acceptable range which is >60%[48,49]. This weaker agreement is a consequence of partial overlapping between the extreme dates from layers 2 and 4; 4 and 5a; and especially 5 and 6. The outlier with the lowest agreement index is a date for layer 5 (Poz-61238, agreement index A=39.7%). The obtained modeled boundaries are provided in Table 3.

Fig 6. Calibrated radiocarbon dates for layers in ShSIII. Dates are arranged here by the order of layers. For details see Table 1. PHA are phases of human activity, described in the section "Chronometric data".

Table 1. Radiocarbon dates for material form ShSIII

Layer	Lab. No.	C-14 date	Material	Inv. No.	Square	Zone	Analytical fraction (atomic C:N ratio for collagen)
1a	Poz-53301	645±30	bone, *Homo sapiens*	W-69	E6	central	collagen (3.00)
A	Poz-56204	730±30	bone, *Homo sapiens*	W-1	D5	central	collagen (3.23)
A	Poz-	2080±30[1]	organic residue	W-382	F7	central	bulk

	56208		in the ceramic vessel				
A	Poz-53302	2190±30[1]	bone, *Ursus arctos*	W-123	E5	central	collagen (2.99)
A	Poz-53300	2270±30[2,4]	bone, *Felis silvestris*	W-93	E5	central	collagen (2.97)
1a	Poz-114532	2430±30	bone, *Felis silvestris*	W-63	D5	central	collagen (3.48)
2	Poz-51326	2810±30[3,4]	bone, *Felis silvestris*	W-183	E6	central	collagen (3.25)
A	Poz-56206	3665±35	bone, *Canis familiaris*	W-239	E6	central	collagen (n.d.)
A	Poz-53308	3745±30	bone, *Canis familiaris*	W-92	E5-6	central	collagen (3.10)
A	Poz-56205	3770±35	bone, *Homo sapiens*	M-98	E6	central	collagen (2.90)
4	Poz-53303	4360±35	bone, *Meles meles*	W-280	E6	central	collagen (3.12)
2	Poz-51327	4475±35[3,4]	bone, *Felis lybica/catus*	W-195	E6	central	collagen (3.26)
4	Poz-53305	4895±35	charcoal	P-40	E6	central	cellulose
4	Poz-61304	4940±35	shell, *Isognomostoma isognomostomos*	-	D-E5-6	central	aragonite
5a	Poz-61305	5870±40	shell, *Isognomostoma isognomostomos*	-	E5	central	aragonite
4	Poz-114530	6160±40	bone, *Felis lybica/catus*	M-6	E6	central	collagen (3.10)
4	Poz-51325	7020±40[4]	bone, *Felis silvestris*	M-8	D6	central	collagen (3.29)
4	Poz-51328	7400±40	bone, *Martes martes*	W-271	E6	central	collagen (3.07)
3a	Poz-61306	7500±40	shell, *Isognomostoma isognomostomos*	-	E4	entrance	aragonite
5	Poz-61237	7570±40	8 shells, *Discus ruderatus*	-	E4-5	central	aragonite
4	Poz-61235	8270±40	3 shells, *Discus ruderatus*	-	D-E5-6	central	aragonite
5a	Poz-61236	8450±40	11 shells, *Discus ruderatus*	-	E5	central	aragonite
6	Poz-61240	8990±40	8 shells, *Discus ruderatus*	-	E5	central	aragonite
5	Poz-61238	9090±50	9 shells, *Discus ruderatus*	-	D-E4-5	central	aragonite
8a	Poz-53307	10,790±70[3]	charcoal	P-62	D6	central	cellulose
8a	Poz-53306	11,000±60	charcoal	P-53	D5	central	cellulose
7b	Poz-	11,730±60	bone, *Lepus* sp.	W-290	D4	entran	collagen (n.d.)

	53304					ce	
8	Poz-61241	11,870±60	3 shells, *Discus ruderatus*	-	D4	central	aragonite
7b	Poz-56207	12,110±60[3]	bone, *Alces alces*	W-455	C5	central	collagen (3.09)

95.4% probability ranges are shown for calibration.
[1] after Sudoł et al.[33]
[2] after Krajcarz et al.[35]
[3] after Sudoł et al.[34]
[4] after Baca et al.[102]
n.d. – not determined due to low collagen yield

Table 2. Optically stimulated infra-red luminescence (IRSL) dates of sediments from ShSIII.

**Layer**	**Lab. No.**	**Annual doze (Gy/ka)**	**Equivalent doze (Gy)**	**Age (ky BP)**	**Inv. No.**	**Square**	**Zone**
5a	Lub-5523	1.37±0.07	44.3±2.4	32.3±2.4	P-TL1	D6	central
6	Lub-5524	2.27±0.11	43.0±1.7	18.9±1.2	P-TL2	D6	central
7b	Lub-5526	2.22±0.11	47.9±1.5	21.6±1.3	P-TL4	D5	central
7b	Lub-5525	2.69±0.13	65.7±2.1	28.9±1.7	P-TL3	D4	entrance

Table 3. The age of the boundaries between phases for ShSIII modelled in OxCal

**Boundaries**	**modelled boundaries (years BP)**		***C* (%)**
	**for 68.2%**	**for 95.4%**	
upper part of the sequence			
upper boundary of PHA 4	670–420	680–-260	97.3
between PHA 3 and PHA 4	2020–650	2050–650	98.5
between PHA 2 and PHA 3	4100–3670	4140–2250	97.6
lower boundary of PHA 2	4310–4020	4800–3990	95.6
lower part of the sequence			
between layers 1a and 2	2870–2470	2920–2400	99.1
between layers 2 and 4	5610–5290	5640–5100	99.6
between layers 4 and 5a	9480–9300	9510–9180	99.6
between layers 5a and 5	10220–9470	10220–9470	98.4
between layers 5 and 6	10240–10180	10250–9980	99.8

between layers 6 and 8*	12730–10180	12740–10180	95.9
between layers 8 and 7b	13950–13700	14060–13610	99.5
lower boundary of layer 7b	14360–13820	16210–13760	95.1

Ages are shown separately for 68.2% and 95.4% probabilities. In case of the lower part of sedimentary sequence (layers 7b, 8, 6, 5, 5a, 4, 2 and 1a), the modelled boundaries represent the boundaries between the natural strata. In case of the upper part (layers 1a and A), the modelled boundaries represent boundaries between the phases of human activity (PHA). *C* is an OxCal measure of convergence.

* no dates were achieved for layer 7a situated between layers 6 and 8, so the modelled chronology of layer 7a falls within this boundary

Malacological succession

The molluskan fauna from ShSIII is composed of 61 taxa of land snails, represented altogether by 10,295 individuals. Fifty-two species are accompanied by 7 taxa identified to the genus level, one to the family level (Clausiliidae), and slug plates represent a collective group of Limacidae (Appendix S2). Malacofauna represent four ecological groups (according to widely accepted subdivision[55]), with 30 shade-loving species (group F), 8 open-country species (group O), 13 mesophilous species (group M) and 1 hygrophilous species *Carychium minimum* (group H). There are only slight differences in malacological composition between the central and entrance zones (Appendix S2), but they differ in proportions between taxa and structure of the assemblages.

No gastropods were found in the lower part of sedimentary sequence (in layers 11, 10 and 9). In the above-lying layers, the mollusk community is bipartite. Only few taxa and up to 60 individuals altogether are noted in layers 7b, 8 and 7a, whereas the higher part of the sequence is characterized by rich malacoceonoses comprising up to 46 taxa and 2181 individuals in a single layer.

Based on both the cluster analysis and the fauna composition, four mollusk assemblages were distinguished in ShSIII. The nominal species are not the most

abundant but rather the most characteristic of the population, which enables wider comparisons on the regional scale[18,103–107].

Assemblage Vt

The sequence starts with the assemblage with *Vallonia tenuilabris* (Vt) (layers 7b, 8, and 7a). This poor malacocoenosis contain from 5 to 60 shells per layer (Fig 7), mostly of open-country gastropods, which constitute 35-73% of all individuals. *Vallonia tenuilabris*, a glacial relic that occupies the open areas of so-called “cold-steppes”, the alpine grasslands, taiga, hemiboreal forest and wooded fens[52,108–111], is the most numerous in the central zone, being accompanied by the shade-loving species *Discus ruderatus* and *Semilimax kotulae*, both characteristic of a cool and continental climate. In the entrance zone, *D. ruderatus* slightly outnumber *V. tenuilabris* (Fig 7).

Fig 7. Paleoecological indicators for ShSIII based on mollusk remains. Data are arranged by the zones and the order of layers. Representation of taxa reflects the percent of individuals. Dark gray bars are used for shade-loving, medium dark for open-country, light gray for mesophilous and white for hygrophilous taxa. “+” means that the taxon is represented in the given layer but the total MNI for the layer is lower than 50, so the representation is not statistical. Numbers in parentheses indicate the number of individuals higher than 1 for statistically non-representative layers. Measures of the paleoecological diversity (richness and Simpson index), representation of habitats and attribution of layers to mollusk assemblages are also shown.

Assemblage DrVc

In layers 6, 5 and 3a, another assemblage dominated by *Discus ruderatus* and *Vallonia costata* (DrVc) can be distinguished (Fig 7). *D. ruderatus* represents taiga-type coniferous forests, whereas *V. costata* is typical of dry and open habitats and predominates in the sequence. In the central zone (layers 6 and 5) glacial relics *V. tenuilabris* and *S. kotulae* are still important components of the assemblage, but they disappear in the entrance zone (layer 3a). Moreover, this assemblage records a first period of predominance of shade-demanding species in the sequence (Fig 7). Mesophilous snails are mostly represented by *Carychium tridentatum*, associated with permanent humid conditions [58] (Appendix S2).

Assemblage DroAp

A rich assemblage with *Discus rotundatus* and *Aegopinella pura* (DroAp) can be distinguished in layers 5a and 4 of the central zone and the lower part of layer 3 in the entrance zone. This assemblage is represented mostly by forest gastropods (52-75% of all individuals) typical of warm and humid conditions, namely, *D. rotundatus*, *A. pura*, *Discus perspectivus, Balea biplicata*, *Helicigona faustina* and *Isognomostoma isognomostomos*. *V. costata* gradually disappears, and the open-country snails become outnumbered by mesophilous species, mainly *Laciniaria plicata* (Fig 7). The occurrence of *V. elata*, unknown in the contemporary fauna of the region, is worth noting.

Assemblage Lp

The topmost part of the sequence (the upper part of layer 3 and layers 2, 1a and 1) is occupied by forest and mesophilous taxa, with the most numerous being *Laciniaria plicata* (Lp). Gastropods of open habitats disappear in the central zone and are of secondary importance in the entrance zone. The forest fauna still comprise a large

fraction of the total fauna (45-61% of all individuals) but are gradually replaced by snails of wide ecological tolerance, which are the most abundant in layers 1a and 1 (up to 55% of all individuals) (Fig 7). *L. plicata*, which is typical of humid rocks in open environments, is accompanied by *Helicigona lapicida*, which often hides in deep crevices in shady rocky substrate; *H. faustina*, which is common in forest slopes and humid shady rocks; and *I. isognomostomos*, which is characteristic of humid mountain forests (Fig 7 and Appendix S2).

Rodent succession

The fossil assemblage includes 1,716 identified specimens, corresponding to a minimum of 901 individuals (sum of MNIs for particular layers) and representing at least 19 taxa, including 17 taxa identified to the species level (Appendix S3). There are no taxonomical differences in the rodent composition between the central and entrance zones. The sediments of the rear zone appeared as very poor in rodent remains and provided only 45 identified specimens in total.

No rodent remains were collected from the lowermost layers (11, 10 and 9). In the upper sequence, two main parts are distinct, which vary in terms of both taxonomic composition and number of specimens (Appendix S3). The lower part (layers 7 and 8) is characterized by a low abundance of rodent remains, low richness and poor diversity (Fig 8). Much richer taphocenosis is preserved in the upper part of the sequence. The number of individuals and number of taxa reach their maxima in layers 5, 5a, 4 and 3 (its lower part). The habitat representation recorded by rodent remains varies clearly between the lower and upper parts of the sequence. The representation of forest environments is low in layers 8 and 7, where the indications of an open tundra environment with the presence of water bodies predominate. The forest component

increases in layer 6 (51%) and continues up to reach over 70% in the upper layers (with maximum in layers 4, 2 and the upper part of layer 3).

The cluster analysis of rodent assemblage from ShSIII reveals four distinct groups, which reflect the distinct faunal assemblages formed by the changes in the composition and structure of the species (Fig 8). These groups were named after the most abundant taxa.

Fig 8. Paleoecological indicators for ShSIII based on rodent remains. The arrangement of figure follows Fig 7. Due to multiple habitats settled by individual taxa, the bars of taxa representation do not indicate the habitats and taxa are arranged systematically (by families and genera).

Assemblage Dg

This assemblage groups the layers 8 and 7b from the central zone. Sublayer 7a and layers 7b-8-7a from the entrance zone possibly also belong to this assemblage. From a quantitative point of view, this is a very poor collection, giving altogether 38 remains from at least 29 individuals. The dominant taxon here is a collared lemming *Dicrostonyx torquatus* (30.8-100% of MNI for sublayers 7a and 7b), followed by the tundra vole *Microtus oeconomus* and European water vole *Arvicola amphibious* (Fig 8). Collared lemming is an inhabitant of cold climatic areas, while the two other voles prefer humid habitats with water bodies. The relatively high representation of the red vole *Clethrionomys glareolus* in layer 8, which is linked with woodlands and prefers densely vegetated clearings, suggests a possible temporal climatic improvement with

development of woodland vegetation. Inhabitants of open dry environments (steppes), i.e., *Cricetus cricetus* and *Sicista subtilis*, were recorded in sublayer 7b.

Assemblage CgMg

Layer 6 forms a separate assemblage. The number of rodent remains is low (51 remains, at least 25 individuals). There is a well-distributed diversity, even if the number of species is low. The assemblage is dominated by *C. glareolus* (28% of MNI). The second-most-abundant species is *Microtus gregalis* (16%), a representative of open and cool habitats. Rodents adapted to cold or boggy environments (*D. torquatus*, *A. amphibious* and *M. oeconomus*) are still present. Thus, the layer records a sensible increase in the woodland component (*Apodemus* and *C. glareolus* constitute 30% of the MNI) (Fig 8).

Assemblage CgMs

A rich assemblage dominated by *C. glareolus* and *M. subterraneus* is recorded in layers 3a, 3 and 1 in the entrance zone, layers 5, 5a and 4 in the central zone, and layers 4 and 1a in the rear zone. Layer 2 from the rear zone is poor in rodent remains but possibly also represents this assemblage. The species of cold habitats disappear. There is a visible rise in the representation of taxa associated with woodland environments, with domination of the red vole, *C. glareolus*. This is the most abundant taxon in the whole upper part of the sequence. Starting from layer 5 in the central zone and 3 in the entrance zone upward, the red vole is followed by *M. subterraneus*, also an inhabitant of woodlands, preferring densely vegetated clearings. Mice of genus *Apodemus* also constitute a significant portion of the assemblage. The development of deciduous or mixed forest habitats is testified by the presence of three species of the Gliridae family, which continues in the upper layers of the sequence (Fig 8).

Assemblage CgMsGg

The layers of the uppermost part of the sequence in the central zone (2 and 1a) are characterized by a separate rodent assemblage. The species representation is similar to the lower assemblage CgMs, and the record of habitat conditions did not change significantly. The visible change is recorded by the higher participation of the edible dormouse *Glis glis*. In layer 1 of the entrance zone and layers 2 and 1a of the rear zone the presence of this species is also marked, which suggests the correlation with CgMsGg assemblage. However, the amount of edible dormouse remains not as high there as in layers 2 and 1a in the central zone, and therefore the cluster analysis joins these layers with the CgMs assemblage (Fig 8 and Appendix S3).

Chiropteran succession

The bat fossil assemblage includes 364 bones corresponding to a minimum of 246 individuals (sum of MNIs of different layers) and representing at least 24 taxa, including 13 species (for the specimens that were identified to the species level) and 6 groups (remains identified to the genus level or belonging to cryptic groups) (Appendix S4); the latter were not considered during the statistical analysis. For some specimens, identification of the species was not possible, since the morphological and dimensional characteristics required for a precise assessment were missing. The groups affected by this issues were *Plecotus auritus/austriacus*, *Pipistrellus pipistrellus/pygmaeus*, *Myotis mystacinus/brandtii/alcathoe/daubentonii*. Some remains (mainly fragmented bones) were identified just to the order level (e.g., Chiroptera).

No bat remains were found in the rear zone. There are differences in the taxonomical composition of bat assemblages between the central and entrance zones. The sediments of the entrance zone are poor in bat remains, with 41 bat specimens representing 10

taxa. The chiropteran remains are much more abundant in the sediments of the central zone, with 144 specimens belonging to 11 taxa. The presence of *Myotis dasycneme* characterizes the entrance zone, while *M. brandtii* occurs in the central zone instead. No bat remains were collected from the lowermost layers (11 to 7). Layers 5a, 4 and 2 yielded the highest number of bat remains (Fig 9). This increase in the presence of bats could be explained by the advance in the forest environment in the area. The cluster analysis of the bat assemblage revealed six distinct groups.

Fig 9. Paleoecological indicators for ShSIII based on bat remains. The arrangement of figure follows Fig 8.

Assemblage PaurMbra

The group comprised by layers 4 and 5a contains the richest assemblage in terms of number of remains, even if the number of species does not differ from the overlying layer 2 (Fig 9). We found 79 individuals belonging to 9 species, for a total of 130 bone remains.

Assemblage MbecPaurMbra

Layer 2 in the central zone seems to be one of the richest layers in terms of a number of remains (NISP = 61, represented by 38 individuals belonging to 9 species). The only fragment attributable to *P. pipistrellus/pygmaeus* (the identification of the species is impossible due to a lack of the distinctive characters) was found in this layer.

Assemblage MdauMbra

This assemblage represents layer 1a from the central zone and is comparable to the one found in layer 1 from the entrance zone. A small number of remains (16 fragments) were found. They belong to 12 individuals of 6 different species (Fig 9).

Assemblage BbarMdas

The lower part of layer 3 from the entrance zone delivered few fragments (16) belonging to 12 individuals of 8 species. This assemblage does not differ considerably from the other layers of the entrance zone (Fig 9).

Assemblage PaurMdauMdas

This assemblage, belonging to the upper part of layer 3 in the entrance zone, includes a small number of specimens (16 bone fragments attributable to 13 individuals of 6 different species) (Fig 9).

Assemblage PaurMdas

This assemblage represents layer 1 from the entrance zone. From a quantitative point of view, this is a very poor collection, with only 16 remains belonging to at least 15 individuals (Fig 9). A predominant taxon cannot be defined.

Discussion

Deposition of sediments

The entire sedimentary sequence in ShSIII can be divided into six sedimentary series (Fig. 10). The differences between series are marked in sedimentary structures (erosional surfaces and lamination), texture (mostly an abundance of limestone clasts) and geochemistry (mostly the amount of organic matter, Ca and P).

Figure 10. Litho-stratigraphic profiles of the Quaternary sediments of ShSIII. Attribution of layers to sedimentary series and correlation between the zones of the site are shown.

Series I – reddish clays

The oldest sediments are preserved in the rear zone as layer 11. This sediment shares similarities with reddish clays known from the lowermost parts of the sequences from caves such as Biśnik (layers 20-23)[10], Nietoperzowa (layer 17)[4], Tunel Wielki (lower part of layer 1)[5], Dziadowa Skała (lower part of layer 1)[112] and Żabia (lower part of sequence)[113]. These sediments were linked with Lower Pleistocene or Pliocene *terra rosa* soils, redeposited to the caves from the surface.

Series II – sands

Layer 10 has also been preserved only in the rear zone. The bright color of sand suggests a correlation with reddish or yellowish sands known from other caves of the region, where they also form a bottom part of the sequences. Such sandy deposits are known from caves such as Nietoperzowa (layer 17)[4], Koziarnia (layer 21)[4], Tunel Wielki Cave (upper part of layer 1)[5], Perspektywiczna (layer 11)[21], Dziadowa Skała (upper part of layer 1)[112] and Wylotne Rockshelter (layer 8)[4]. The accumulation of sands may be connected with Middle Pleistocene glaciations, when the area of Kraków-Częstochowa Upland entered the fluvioglacial regime[26,27,114], or with later colluvial redeposition of fluvioglacial sediment. Both layers 11 and 10 fill the narrow depression of the bedrock, a form which was called a "bottom rill"[115] and which is most likely the remnant of a vadose canyon. This depression was also noticed in the central zone, but

here, the sediments resembling layers 10 or 11 have not been preserved. The depression is filled with limestone debris of layer 9, which also occurs in the rear zone, but with limited thickness and at higher elevation. This result indicates that erosional event happened between accumulation of layers 10 and 9, which was responsible for almost total removal of layers 10 and 11, except for the rear zone.

Series III – limestone debris and loess

Layer 9 records the cold climatic conditions. A high amount of angular limestone clasts with Ca-rich silty matrix indicates that physical weathering of the bedrock and consequential rockfall was the most important depositional factor, followed by physical weathering of the rockfall debris. An absolute lack of faunal remains additionally suggests very cold periglacial conditions. This sediment may be correlated with layer III from the nearby Shelter above the Zegar Cave[116], described as loess with limestone debris, with very sparse faunal remains. The reddish color of layer 9 suggests that layer 11 (and possibly also layer 10) served as an additional source of material for layer 9. Most probably, some remnants of layers 11 and 10 survived the erosion adhered to the walls or inside the niches and dropped down during the deposition of layer 9. A similar situation concerning the Lower Pleistocene red clays redeposited into the Upper Pleistocene series was described for Tunel Wielki Cave[5,13]. Debris of layer 9 filled the deep vadose canyon cleaned by the previous erosion and achieved over 1 m thickness inside the depression, but it reached only limited thickness on the elevations, such as in the rear zone.

Layer 7 was deposited on the uneven surface of the rockfall debris and filled the depressions. The layer achieves the greatest thickness near the entrance and becomes thinner toward the cave interior. It disappears at a distance of approximately 300 cm from the dripline. It is a loess-like sediment or "cave loess"[11]. Respecting the

lithostratigraphic scheme of those authors, the lower part of layer 7 (with limestone debris) correlates with their unit A, and its upper part (less debris, more silt) with unit C. Analogous sediments occur in a number of caves in the region, including the closest ones: Biśnik Cave and Shelter above the Zegar Cave[10,116]. Indistinct lower boundary with layer 9 indicates gradual change in sedimentary environment from physical weathering related to cold and humid climate to eolian accumulation typical for cold and dry conditions.

Intercalation of dark-colored layer 8 allows dividing layer 7 into sublayers 7a and 7b, but this possibility occurs on a limited area. Sublayer 8a in the lower part of layer 8 represents an anthropogenic activity. This deposit was formed by firing a hearth in a shallow pit dug in the loess. Few faunal remains have been found in layer 8. These include both the bones of larger mammals (hare and elk), which may be related to human hunting activity but also the remains of rodents and snails, likely deposited by natural agents. Presence of fauna and charcoals records a temporal climatic warming, replaced later again by periglacial conditions as marked by the overlying loess of layer 7a. Trough cross-stratification visible in sublayer 7a also indicates a role of colluvial processes in the formation of this stratum.

Series IV – colluvial sediments

Holocene sequence starts from layer 6, as indicated by radiocarbon dating (Table 1) and explosion of the abundance and diversity of faunal remains (Fig 11). Trough cross-stratification and lamination in layers 6 and 5 are distinct indicators of erosion and colluvial redeposition of sediments by washing and/or mudflows. Inclination of sedimentary structures indicates that the main direction of a geological transport was oriented from the rear zone of the rockshelter to the entrance and probably outside. Redeposited material differs from any lower sediment by the higher amount of organic

matter, Ca and P. This result means that before the redeposition, but after the sedimentation of layer 7, another type of sediment was accumulated in the rockshelter. The original strata did not survive the denudation, but their material has been preserved in a form of layers 6 and 5. The source of this material was most likely situated in the rear zone.

This series represents a period of geomorphological instability, when episodes of erosion and colluvial redeposition alternated several times in the rockshelter. The reason for these processes was high water supply, possibly linked with humid climate or degradation of the Pleistocene permafrost. The series is topped with an unconformity, which may be regarded as the last record of erosional-colluvial activity. Records of analogous processes were recognized in other cave sites in the region [11] and linked with the lithostratigraphic unit E of the loess-like cave deposits.

Fig 11. Summary distribution of radiocarbon probabilities for ShSIII. Calibrated dates are used. PHA are phases of human activity, described in the section "Chronometric data".

Series V – massive loams

Structureless (massive) silts and loams with limestone debris of layers 5a, 4, 3a, 3 and 2 represent separate series. No sedimentary macrostructures except for interlayer boundaries are visible, and even these boundaries are indistinct and gradual. This indicates a low sedimentation rate and slow or almost ceased geological transport. High amount of limestone clasts and high Ca content in the fine fraction support a hypothesis of an autochthonous source of the clastic material. A high concentration of P indicates

intense zoogenic accumulation of excrements of cave dwelling animals and/or carcasses of their prey. The limestone clasts are smoothed and even rounded-like. Madeyska[13] linked the smoothing process with both mechanical erosion and chemical dissolution by dripping water and a generally humid and warm climate[38]. Layers 5a and 4 are particularly distinct due to their bright color and higher amount of clay. Increased content and yellowish color indicate chemical weathering of limestone, i.e., the dissolution and removal of carbonates, accompanied by deposition of water-insoluble clay residuum enriched in Fe oxides. The bright color and higher amount of clay in cave sediments are regarded as an indicator of warm climate [31] and allows correlating of these layers with the Holocene climatic optimum.

In the entrance zone, the stratigraphy is less distinct due to pedogenesis (see the section "Pedogenesis" below). Noteworthy is the presence of large limestone blocks in layer 3. Some of them reach more than 90 cm in diameter. These blocks represent a massive rockfall event. Other large blocks (some of them longer than 1.5 m) protrude from the ground at the plateau in front of the rockshelter and possibly represent the same episode. As the excavation area uncovered only a limited part of this accumulation of blocks, it is difficult to conclude regarding its origin. It could represent the climatic conditions favoring physical weathering and disintegration of rock, i.e., a cold climate, but this is in contradiction with the record of a climatic optimum within the series. An alternative explanation is a catastrophic event, such as an accidental fall of a heavy block from the upper part of the hill, which could cause a massive avalanche.

Series VI – dark humus-rich top sediments

The uppermost layers 1 and 1a differ from sediments of the underlying series V. The most impressive feature is their dark color, related to increased humus content. The sand fraction is clearly higher. The unusually high concentrations of Pb and other toxic

elements (Ag, Bi, Cd, Sb, Sn, Zn) suggest an increased impact of human activity. However, no archaeological artifacts made from these metals were found in the rockshelter. This suggests indirect pollution from the outside. It may be noteworthy that lead, zinc and silver mining in the southern part of Kraków-Częstochowa Upland has been active at least since Middle Ages[117] and is still ongoing. This medieval and modern industry could have polluted the region, e.g., via an eolian activity. Sediments of this series contain detrital material, i.e., bones with quite early radiocarbon dates (Fig. 6). These bones likely come from the backfill of archaeological feature, layer A, and testify later disturbance of the grave, possibly by burrowing animals.

Postsedimentary disturbances

The sediments of ShSIII are relatively intact. Cryoturbation and bioturbation have had limited importance and affected only narrow stratigraphic horizons (Fig. 10). The most extensive disturbances are those produced by pedogenesis and human activity.

Cryoturbation

Although the thick series of sediments were connected with cold climate (layers 7 and 9), the traces of cryoturbation are restricted in the sedimentary profile. The only distinct disturbances caused by frost action are visible in layers 10 and 11. A complex of these layers together with frost structures are cut by the unconformity and covered by layer 9. This indicates that the cryoturbation event took place before the deposition of layer 9 and the preceding erosion.

Bioturbation

Larger fossil burrows were found inside layers 5a and 5. The size of burrows suggests a constructor of a badger or fox size. These structures were filled with a sediment resembling layer 4. A badger bone found inside one burrow gave a

radiocarbon age of 5.0-4.8 ky BP (Table 1). Some further dates obtained for layers 5a and 5 (Poz-61237 and Poz-61305) are consistent with dating of layer 4 (Table 1). These data allow correlating the burrowing activity with the deposition of layer 4 or a period soon after.

The presence of a brown bear skeleton[33] suggests that the rockshelter was used by bears as a hibernation den. It cannot be excluded that bears dug the hibernation pits and disturbed the sediments. However, the bones of a bear were found in a secondary position inside the backfill of an anthropogenic feature (layer A). The direct relationships between the bear remains and the sediments cannot be reconstructed.

An interesting example of the recent bioturbation are funnel-shape pits in the modern ground surface, dug in the layer 1a and filled with excrements, possibly of a badger. Two such structures were found during excavation, each approximately 20 cm deep, approximately 20-30 cm wide in the upper diameter and narrowing downward. The total volume of excavated excrements was approximately 10-12 liters. These pits can be interpreted as animal latrines. Production and use of such communal latrines is a known behavior of badgers[118,119]. Such badger activity might be responsible for the lateral scattering of some material from the backfill of the grave (layer A) throughout the rockshelter, and its incorporation into layer 1a.

Anthropogenic disturbances

The first sediment-disturbing human activity in the rockshelter was related to PHA 1 and happened during the Late Paleolithic. It affected the loess layers 7b and 8 in the area close to the rockshelter entrance. The Paleolithic visitors dug an oval pit that served as a fireplace. This trough-like object had a diameter of 60 cm and a depth of 30 cm and must have been filled with loess sediment (layer 8a) soon after humans had left the site.

The second distinctive phase of the anthropogenic disturbances is related to the burial of a woman in the late Middle Ages (PHA 4). The grave was constructed in the central zone and covered an area of approximately 2 m^2 (at squares D/5, D/6, E/5, E/6 and E/7). The length along NE-SW line is approximately 200 cm, whereas the width along NW-SE line is approximately 100 cm, and the greatest depth reaches 60 cm. The object contacts the limestone walls of the rockshelter (the bedrock) from the west and south-west, while several larger stones that can be interpreted as a relic of a purposefully built wall have been found in the entrance zone, at the north side of the grave[33]. The boundaries of the grave cut primarily layers 1, 1a, 2 and 3 and partially layers 4 and 5a in the southern part. The object is filled with sediments named layer A (field numeration "1a/2" and such name was used in a previous paper [32]). Inside the entire backfill of the object, coal dust and fine charcoals were dispersed. Lithologically, the backfill resembles layers 1, 1a and 2, whose material was probably used to fill the grave. The backfill does not exhibit any visible stratification. This suggests a quick and single-event deposition of the sediment. Toward the bottom, the sediment becomes lighter in color and more clayey, similar to layer 4. This is probably a result of using the redeposited sediment of layer 4 during the initial filling. Archaeological material within the object is associated with different phases of human activity (PHA 2, PHA 3, PHA 4, see Fig 11). It is mixed up and does not exhibit any systematic layout, except of human bones linked to PHA 4. This indicates that medieval people destroyed the earlier objects and used their backfills while constructing the grave. It seems probable that other features (perhaps graves or pits) associated with PHA 2 and PHA 3 were present in the central zone before the Middle Ages. However, the construction of the medieval grave completely consumed these older objects.

Pedogenesis

In the entrance zone, the stratigraphy of the upper part of the sequence is obscured by dark coloration, related to a high content of organic matter. The dark color becomes gradually lighter downward from the top of layer 1 to the bottom of layer 3, which is also marked by the gradual decrease in organic matter (Fig 5). This indicates that the organic matter was dispersed in profile by illuviation. Layer 1 represents an A-horizon (topsoil) and layer 3 represents a B-horizon (cambic) of a cambisol. The development of the soil was (and still is) possible here due to localization outside of the cave, as layers 1 and 3 occur only in the entrance zone, close to the dripline or outside of it. Recently, this area has been covered by forest litter composed mostly of beech and oak leaves (Fig 2).

Layer 3a possibly represents a separate, earlier phase of pedogenesis. This stage of soil development was possibly halted by the erosion recorded in the bottom of series V and/or the accumulation of large limestone blocks visible now inside layer 3 (see the section “Series V – massive loams” and Fig. 4). Some strata situated inside the rockshelter, namely, layers 1a and A and to a lesser extent layer 2, are also dark-colored and enriched in organic matter. This enrichment can be an effect of occasional input of litter to the interior of the rockshelter or redeposition of the humiferous sediment of layer 1 from the entrance zone (or topsoil material from outside the rockshelter) by wind, sheet wash and/or walking animals and humans.

Age model

Chronological frame of the sequence

The radiocarbon and IRSL dates indicate the Late Pleistocene and Holocene chronology of the sediments in ShSIII. Dates arranged by layers show a consequent chronological order (Fig 6). The only inconsistency that prevented the OxCal sequence

model from running (two dates were removed from the model; see the section "Chronometric data") is connected with layers 4-5a-5. It is noteworthy that these layers were a subject of bioturbation, as burrows filled with sediments of layer 4 were detected in layers 5a and 5 (see the section "Bioturbation"). That burrowing could be responsible for mixing of the subfossil material and observed chronological disorder. Two dates removed from the model (Poz-61237 and Poz-61305) were achieved on snail shells excavated from layers 5a and 5 but fit well to the set of dates for layer 4. These two dates likely represent intrusive material according to the terminology of Bronk Ramsey[49], in this case material from layer 4 that had been redeposited to the lower layers through animal burrows.

Pleistocene sediments

No dates are available for the lowermost layers 9, 10 and 11. The chronology of these layers can be approximated as being older than the earliest date accessible for the upper strata, which is 28.9 ± 1.7 ky BP (Table 3). Layers 7b and 8 clearly represent the Late Pleistocene. The radiocarbon dates fall between 14.5 and 12.5 cal ky BP for both layers. Bayesian modeling estimates the age of lower boundary of this series to be between 16.2 and 13.8 ky cal BP with 95.4% probability (see Table 3 and the section "Chronometric data"). The IRSL dates indicate much older ages, approximately 21-28 ky cal BP. These dates likely represent a periglacial conditions of loess accumulation, when biogenic material was not deposited. No dates are available for layer 7a. The modeled statistical age of this layer is between 12.7 and 10.2 ky cal BP (with 95.4% probability; see Table 3 and the section "Chronometric data") and correlates with the Younger Dryas interstadial and/or Preboreal phase of the Early Holocene[120].

Holocene sediments

Radiocarbon dates indicate almost continuous accumulation of biogenic material through entire Holocene (Fig 11). The greatest gap in the probability distribution of radiocarbon age falls before 10.5 ky cal BP, i.e., in the beginning of Holocene.

Series IV (layers 5 and 6) includes the oldest Holocene sediments. The series was deposited during relatively short time interval according to radiocarbon dates, which lasted for several hundred up to a maximum of three thousand years (Table 3). The IRSL date ~~achieve~~d for layer 6 shows ~~a~~ much older age of clastic material than radiocarbon dating of animal remains (Table 2) and corresponds with the age of layer 7b. Regarding the colluvial origin of the series, this date can likely indicate a colluvial redeposition of the material of layer 7b, which has been incorporated into layer 6. A relatively early age is also indicated by the IRSL date for layer 5a. This age is earlier than any other date obtained for layer 7b and may possibly record another source of material (layer 9 or sediments occurring outside the rockshelter?) It is difficult to conclude regarding the sedimentary process responsible for accumulation of this material. No macroscopic traces of colluvial processes were identified. The important role of zoogenic deposition (see the section “Series V – massive loams”) suggests that deposition of dust or mud by walking and trampling animals could be one of the accumulation processes.

The longest time interval is recorded by layer 4. Its deposition lasted for 4-5 thousand years according to the Bayesian model (Table 3). This interval corresponds to the late part of the Early Holocene and the entire Middle Holocene according to the stratigraphic subdivision[121]. It also corresponds to the entire Atlantic climatic chronozone[120]. Layer 3a from the entrance zone correlates with layer 4 according to the dating results.

Layer 2 corresponds with Late Holocene. Poor chronometric data are available for layers 1-1a. However, assuming that the archaeological feature corresponding to PHA 4 cuts these layers and the lower boundary of PHA 4 was estimated to be 2.0-0.7 ky cal BP (Table 3), the accumulation of layers 1-1a started before that phase. This dating is in good agreement with the modeled upper boundary of layer 2, which is 2.9-2.4 ky cal BP (Table 3).

Paleoecology and reconstruction of paleoenvironment

The abundance of fossil material makes the ShSIII an important paleontological site. Although Holocene paleofaunas were reported from other caves of southern Poland and adjacent countries, only few of these sequences go back as far as the final part of the Last Glaciation[17,18,122–127]. Most of the profiles are only a few dozens of centimeters thick and cover only the middle or just the youngest part of the Holocene[103,104,128–132]. Despite some disturbances noted in the narrow stratigraphic horizons in the ShSIII, the composition of the mollusk and rodent faunas appears to be environmentally coherent, which makes both groups good paleoenvironmental indicators.

Reliability of paleoenvironmental reconstruction

The mollusk thanatocoenoses in caves represent well the diversity of habitats in the surroundings, comprising shells of species penetrating the cave, of those living on the surface near the cave entrance and the shells dropped from the surrounding rocks during sedimentation[133,134]. The rather good state of preservation of shells in ShSIII suggests that they did not undergo any intensive transportation but rather were accumulated in sediments in the primary place of deposition after the mollusk death.

The rodent remains were deposited mostly from pellets dropped by carnivorous birds, such as owls resting in a cave[135]. Rodents likely could have inhabited a range of

habitats surrounding the cave, which had been used by the birds as their hunting territories. Therefore, rodents represent a wider area than mollusks. It is also expected that predatory birds prefer open habitats over canopy for use as hunting grounds[136,137], so as a consequence, the abundance of open-habitat rodents can be overrepresented in the fossil record.

Bats are unique among the fossil fauna, as they are troglophiles who live inside caves. Some of the recorded taxa are known to hibernate in caves[138], while the others use caves as their summer roosts[70]. Bats are also migrants and some species move between their winter and summer areas at the distance of over hundred kilometers. Thus, species who used the caves as their hibernacula do not represent the habitats surrounding the cave. This may be the case for *M. dasycneme*. This species is known to hibernate in caves[138], so its presence in ShSIII is possibly an effect of death during winter. The water bodies used by this species as hunting grounds[100,101] were located in its summer area, which could be far from the site.

Final Pleistocene

The oldest assemblages of mollusks and rodents (Vt and Dg) found in layers 7b-8-7a represent a rather poor fauna. Both assemblages are composed of species of cold climate, known from tundra and steppe biomes. Cold-tolerant snails *Vallonia tenuilabris* and *Discus ruderatus* indicate a cool continental climate and predominance of open habitats around the cave. The same habitats are preferred by lemmings. Dry open environments, i.e., steppe, are also indicated by the presence of *C. cricetus* and *S. subtilis*. However, some patches of cool, humid and shady habitats favorable for *S. kotulae*[58] also occurred, which indicates a mosaic of habitats. The rough and hilly topography of Kraków-Częstochowa Upland certainly was a factor favoriting the diversity of habitats. This is confirmed by regional paleobotanical data[139], which

indicate that during Late Glacial of the Last Glaciation the clusters of trees dominated by pine, spruce and larch occurred beside the rocky slopes occupied by steppe-like communities.

The most informative is layer 8 with the highest number of shells. The occurrence of *S. kotulae* and *D. ruderatus* and lack of species typical of arctic climate, e.g., *Pupilla loessica*, *Vertigo genesii* and *Vertigo geyeri*, may indicate Bølling-Allerød interstadial[105,140]. Similar malacofaunas of this age are known from calcareous tufas and landslide deposits of southern Poland[130,131,140–142]. The rodent remains, though still low in number, follow the mollusk trend. They document higher abundance of woodland and humid environments during deposition of layer 8 in comparison to layers 7a and 7b. Additionally, the first appearance of a woodland species *C. glareolus* is recorded in layer 8.

Pleistocene-Holocene transition

Rich and diverse mollusk fauna of DrVc assemblage points to the beginning of the Holocene and amelioration of the climatic conditions compared to the Late Glacial. Radiocarbon dating of layers 6 and 5 from the central zone and 3a from the entrance zone indicate the Preboreal and Boreal phases of Early Holocene, and possibly the very beginning of the Atlantic phase [120]. The mollusk composition resembles the *Ruderatus*-fauna[143] known across Europe from many terrestrial sequences between the Preboreal and the older part of the Atlantic phase[52,54,146–150,105–107,126,127,140,144,145]. Expansion of shade-loving taxa, namely, *Discus ruderatus*, suggests growth of taiga-type coniferous forests, but some grasslands and sunny slopes preferred by *Vallonia costata* also occurred around the site. If shells have not been reworked, the environmental conditions in the beginning of Holocene favored the longer occurrence of glacial relics *Semilimax kotulae* and *Vallonia tenuilabris*. The last one was noted in Preboreal deposits in the

nearby Cave above the Słupska Gate[18]. Gradual disappearance of glacial relics may correspond to gradual climate warming and some oceanic influences inferred from the first appearance of *Sphyradium doliolum*, *Discus rotundatus*, *Aegopinella pura* and other species of warm and humid conditions[18] (Fig 7).

The rodent assemblage CgMg found in layer 6 is still not abundant; however, it exhibits the highest Simpson index in the sequence. This reflects the high diversity of species and of habitats, as both the species of forest and of open landscape occur. Like in the case of snails, the rodent glacial relics survived, namely, *Dicrostonyx torquatus* and *Lasiopodomys gregalis*. According to Nadachowski[137], the glacial relics could survive in the region until the beginning of Late Holocene. Layer 6 is marked by the first appearance of *M. subterraneu*s and genus *Apodemus* in the sequence. The first one was found in Late Glacial deposits of Shelter above the Niedostępna Cave[5,151], which is situated approximately 26 km away. In ShSIII this species is lacking in strata of that age and becomes abundant starting from layer 5 upward. This observation suggests reconsidering the early appearance of *M. subterraneus* in Shelter above the Niedostępna Cave, which could be an effect of postdepositional reworking. In another site with well-preserved Holocene deposits, Nad Mosurem Starym Duża Cave, its first appearance is even later, estimated to be ca. 4 ky BP[17].

Rodents of layer 5 record rapid improvement of environmental conditions. This is reflected by the increase of the remain number and higher amount of forest dwellers, such as *C. glareolus*, red squirrel *Sciurus vulgaris* and glirids *Eliomys quercinus* and *Muscardinus avellanarius*.

Interesting issue is a stratigraphic (and time) shift between the start of dominance of forest fauna in the mollusk and rodent records. The pick of forest-dwelling mollusks begins in layer 6 (or in layer 3a in the entrance zone), while in the case of rodents, only

in layer 5 (or 3 in the entrance zone). This situation may be an effect of taphonomic biasing. As the accumulation of rodents prefers the taxa of open landscape (see the section "Reliability of paleoenvironmental reconstruction"), the dominance of forest rodents in the taphocenosis could have been intense since the forested area was virtually the only habitats in the surroundings, although patches of forest inhabited by forest rodents had been abundant earlier. The lack of chiropteran remains in the lower layers and their scarcity in layers 6 and 5 may be an effect of the unfavorable environmental conditions.

Holocene climatic optimum

Predominance of mixed and deciduous forests around the cave during sedimentation of layers 5a, 4 and 3 is evidenced by DroAp mollusk assemblage, CgMs rodent assemblage, PaurMbra chiropteran assemblage and radiocarbon dating (9.5-4.5 ky BP). *Discus ruderatus* was replaced by more demanding *D. rotundatus*, *D. perspectivus* and other strictly forest species, e.g., *A. pura*, *Balea biplicata*, *Ruthenica filograna* and *Isognomostoma isognomostomos* (Fig 7). This records the progressive warming and was likely connected with transition from continental to oceanic circulation across Central Europe and the climatic optimum of the Holocene – the Atlantic phase[52,106,107,145,148,152–154]. These data are in agreement with floristic changes in Central Europe[155,156]. The climatic optimum is indicated by the presence of *Vestia elata*, which has narrow ecological tolerance and favors warm and humid conditions. This snail has been described from Atlantic phase at other sites in the region and disappeared during the Subboreal phase[122,123,157]. Today, only limited relict sites with this species are noted in Poland in the Beskidy, Bieszczady and the Holy Cross Mountains[158].

The composition of rodent taphocenosis in layers 5a, 4 and 3 is similar to that in layer 5. All these layers are characterized by the highest richness of taxa (Fig 8). The

important difference in comparison to layer 5 is much higher number of remains. Simpson index of diversity is lower than in most of other layers, which is a result of strong predominance of two species, *C. glareolus* and *M. subterraneus,* both typical for woodlands.

The chiropteran assemblage PaurMbra, found in these layers, is also among of the richest in the sequence, both in terms of number of taxa and number of remains. The thanatocoenosis is characterized by taxa which prefer cold caves as their hibernacula, with temperature below 6°C (which stays in accordance with small size of the ShSIII and testifies that the shelter already had than a similar size to the modern one), and big trees as summer roosts (*B. barbastellus*, *M. bechsteinii* and *P. auritus*). This suggests the presence of old-growth forest with sparse open areas (because of the presence of *Eptesicus serotinus*) The occurrence of *Rhinolophus hipposideros*, a species that typically prefers warmer caves to hibernate, could be limited to the summer period.

Post-optimum deterioration of habitats

Starting from approximately 5 ky BP, the malacofauna of ShSIII are dominated by mesophilous species. The forest malacofauna are still diverse but significantly outnumbered, mostly by *Laciniaria plicata* (Fig 7). The overall tendency of an increasing number of gastropods of wide ecological tolerance in costs of shade-loving taxa suggests a gradual deforestation and exposure of rocky walls around the cave. A similar situation is recorded in other Subboreal and Subatlantic sequences from caves and rockshelters of Central Europe[18,103,104,126,127,145].

The upper part of the sequence is characterized by a high abundance of edible dormouse *Glis glis*, which is the third most numerous rodent species in layers 2 and 1a. Its abundance probably coincided with a change in forest cover around the cave and rise

in mature beech forest, which is a preferred habitat of this species[77,80]. Beech forest also constitutes the main type of the recent natural vegetation in the microregion. The expansion of this tree into the region was quite late, the beech forests appeared here approximately 3.5 ky BP[159]. This dating corresponds well with the presumed chronology of the mentioned layers.

The uppermost layers 1 and 1a record also the first appearance of *M. arvalis* and *Micromys minutus*. The arrival of both these species was most likely of synanthropic character, as they prefer artificial landscapes (agricultural fields and meadows). A similar observation of the appearance of *M. arvalis* during the late part of Late Holocene was noticed from other sites in the region[137]. Particularly, the Holocene profile of Nad Mosurem Starym Duża Cave shows the same pattern of rodent succession[17]. The presence of single remains of tundra species (*Dicrostonyx torquatus*) in layer 1a may be an effect of post-depositional disturbances.

Chiropteran fauna notes decline in richness and number of specimens, following the trend in mollusks and rodents. There is a clear predominance of taxa foraging in wooded areas (i.e., *B. barbastellus*, *M. bechsteinii*, *P. auratus* and *M. brandtii*). The presence of *M. myotis* can be related to a lack of undergrowth in the surrounding forest, while *M. mystacinus*, *M. nattereri*, *M. emarginatus* and *P. austriacus* suggest the presence of open and/or diversified areas, too. All these data likely indicate deterioration of habitats, most likely of anthropogenic origin.

Chronology of human activity

Four episodes related to human activity have been recorded at ShSIII (see Fig 11). The trace of the oldest is the relic of a Late Paleolithic hearth (PHA 1), probably related to the short-term camp[34]. Another episode (PHA 2) is associated with a possible burial

containing human and animal remains, which has been fragmentarily preserved in the secondary deposit inside the backfill of the medieval grave, and whose radiometric dating implies the activity of an Early Bronze Age community. The next phase of human stay (PHA 3) happened in the late pre-Roman period. Fragments of the vessel[33] and the animal deposit[35] were all redeposited into the medieval grave. Their context and nature imply the sepulchral character of human activity from 4th to 1st century BC. Typology of the vessel based on stylistics and comparison with the other sites indicate the second half of the 1st century BC. Radiocarbon dating of the residue collected from the inner surface of the vessel fragment and of the wildcat mandible with cut marks indicate somehow earlier chronology, between the 4th and 1st centuries BC[33]. The youngest phase of human activity in the shelter (PHA 4) is a late Middle Age burial.

The cultural episodes identified at the rockshelter have numerous analogies in the nearest area. The Early Bronze Age sites are common in the caves of the region. The archaeological material (mainly pottery and flint artifacts associated with the production of tetrahedral axes) indicates that human activity in the other caves in the region was limited primarily to the plateaus outside of the caves or elevated places over the caves[20,116,160–163]. During the late pre-Roman period, the human activity in the caves of the Kraków-Częstochowa Upland increased. Ceramic vessels are the most common artifacts in other sites, but coins, elements of belts, fibulae and toiletry objects have also been found. Typically, the archaeological inventories suggest the economic and utilitarian meaning of caves for those people[164,165]. It should be emphasized that the nature of deposits in the ShSIII, i.e., a buried vessel and a sepulchral deposit of the European wildcat (*Felis silvestris*), make them unique among the Celtic sites in the region[33].

There are many examples of human activity in the caves of Kraków-Częstochowa Upland during the Middle Ages[1]. It is highly probable that the medieval burial in ShSIII could be related to the medieval castle Smoleń situated on a neighboring hill and the nearby village Pilica. However, the mysterious fact is a human burial inside a cave. This happened several centuries after the Christianization of the region, when cemeteries were commonly used as burial places.

Considering the small space of the rockshelter, the multicultural deposits and their sepulchral nature indicate the supermaterial significance of the place from Antiquity to the Middle Ages. It seems that the rockshelter has been visited for sacral or magical purposes[33].

Conclusions

The almost two meters-thick sedimentary sequence of Late Glacial and Holocene sediments in ShSIII is unique among the caves of the Kraków-Częstochowa Upland and the caves of Central Europe in general. Although Upper Holocene cave sediments are quite common in the southern Poland[16,17,128,166], the Lower Holocene is preserved only on very rare occasions. In case of ShSIII, the entire profile of Lower-Middle-Upper Holocene is preserved, which is an exceptional situation. The additional value of this site is that its stratified lithostratigraphic sequence contents an abundant paleozoological material. Cave sites with long profiles of subfossil fauna of mollusks, rodents and chiropterans are unique for Central Europe and present a great value for reconstructions of paleoecology in the region. Taking all these facts into consideration, we propose to regard ShSIII as a regional stratigraphic stratotype of Holocene clastic cave sediments.

The synthetic stratigraphy of ShSIII is presented in Fig 12. The lithology and geochemistry of sediments allow reconstruction the depositional conditions and can be regarded as a reference for future studies of cave sediments in the region. The composition and structure of the mollusk and rodent assemblages and to lesser extent also chiropterans, allow reconstructing the changes in paleofauna, which reflected the changes of climate and vegetation around the cave.

Because ShSIII is a small cave, the archaeological excavations consumed the greater part of its sediments (Fig 3). After the excavations, the profiles of unexcavated sections have been protected and the archaeological pits carefully backfilled with stones and previously sieved sediment. This makes the site secured and ready for eventual re-excavation in future, including sampling for further analyses. However, its unique scientific value makes it especially important to elaborate the project of legal protection for this site. The initial actions have already been made, as the surroundings of the site are included in the 'Natura 2000' protected area (*Ostoja Środkowojurajska*, site code PLH240009). Despite this, further particular protection of the cave is needed, especially in terms of the future scientific research and access to secured profiles.

Figure 12. Stratigraphy of the sediments from ShSIII and reconstruction of the paleoenvironmental conditions. Boundaries of the geochronological units after Cohen et al.[121] and boundaries of the climatic zones after Starkel et al.[120].

Acknowledgments

We are thankful to the officers of the Polish National Forests for their help with the organization of excavations; and to the students of archaeology from Nicolaus Copernicus University in Toruń who participated in the excavation works.

References

1. Wojenka M. Caves of the Kraków-Częstochowa Upland in the Middle Ages. An introduction to the issues. Prądnik Pr i Mater Muz im Prof Władysława Szafera. 2012;22:7–43.
2. Wojenka M. Caves in the Post-medieval landscape of the Polish Jura Chain. In: Valde-Nowak P, Sobczyk K, Nowak M, Źrałka J, editors. ultas per gentes et multa per saecula. Kraków: Jagiellonian University in Kraków; 2018. p. 585–92.
3. Szelerewicz M, Górny A. Jaskinie Wyżyny Krakowsko-Wieluńskiej. Warszawa-Kraków: Wydawnictwo PTTK „Kraj"; 1986.
4. Madeyska-Niklewska T. Górnoplejstoceńskie osady jaskiń Wyżyny Krakowskiej. Acta Geol Pol. 1969;19(2):341–90.
5. Madeyska T. Osady jaskiń i schronisk Doliny Sąspowskiej. In: Chmielewski W, editor. Jaskinie Doliny Sąspowskiej Tło przyrodnicze osadnictwa pradziejowego. Warszawa: „Prace Instytutu Archeologii UW", Wyd. Uniwersytet Warszawski; 1988. p. 77–173.
6. Madeyska T. Stratigraphy of the sediments in the Cave Mamutowa at Wierzchowie near Cracow. Folia Quat. 1992;63:35–42.
7. Cyrek K, Nadachowski A, Madeyska T, Bocheński Z, Tomek T, Wojtal P, et al. Excavation in the Deszczowa Cave (Kroczyckie Rocks, Częstochowa Upland,

Central Poland). Folia Quat. 2000;71:5–84.

8. Cyrek K, Madeyska T. Cave fillings - A chronicle of the past. An outline of the Younger Pleistocene cave sediments study in Poland. Acta Geol Pol. 2002;52(1):75–95.

9. Nadachowski A, Żarski M, Urbanowski M, Wojtal P, Miękina B, Lipecki G, et al. Late Pleistocene Environment of the Częstochowa Upland (Poland) Reconstructed on the Basis of Faunistic Evidence from Archaeological Cave Sites. Kraków: Institute of Systematics and Evolution of Animals, Polish Academy of Sciences; 2009.

10. Krajcarz MT, Bosák P, Šlechta S, Pruner P, Komar M, Dresler J, et al. Sediments of Biśnik Cave (Poland): Lithology and stratigraphy of the Middle Palaeolithic site. Quat Int. 2014;326–327:6–19.

11. Krajcarz MT, Cyrek K, Krajcarz M, Mroczek P, Sudoł M, Szymanek M, et al. Loess in a cave: Lithostratigraphic and correlative value of loess and loess-like layers in caves from the Kraków-Czestochowa Upland (Poland). Quat Int. 2016 Apr 18;399:13–30.

12. Valde-Nowak P, Alex B, Ginter B, Krajcarz MT, Madeyska T, Miekina B, et al. Middle Paleolithic sequences of the Ciemna Cave (Prądnik valley, Poland): The problem of synchronization. Quat Int. 2014;326–327:125–45.

13. Madeyska T. Środowisko człowieka w środkowym i górnym paleolicie na ziemiach polskich w świetle badań geologicznych. Stud Geol Pol. 1981;69:1–125.

14. Madeyska T. Osady schroniska w Kruczej Skale w Skałach Kroczyckich (środkowa część Jury Polskiej). Kras i Speleol. 1996;8(17):57–65.

15. Krajcarz MT, Krajcarz M, Goslar T, Nadachowski A. The first radiocarbon dated steppe polecat (Mustela eversmanii) from the Pleistocene of Poland. Quat Int. 2015;357:237–44.

16. Kowalski K, Kozłowski J, Krysowska-Iwaszkiewicz M, Pawlikowa B, Wiktor A. Badania osadów schronisk podskalnych w Żytniej Skale (Bębło, pow. Kraków). Folia Quat. 1967;25:1–27.

17. Nadachowski A, Madeyska T, Rook E, Rzebik-Kowalska B, Stworzewicz E, Szyndlar Z, et al. Holocene snail and vertebrate fauna from Nad Mosurem Starym Duża Cave (Grodzisko near Cracow): palaeoclimatic and palaeoenvironmental reconstructions. Acta Zool Cracoviensia. 1989;32:487–511.

18. Szymanek M, Krajcarz M, Krajcarz M, Alexandrowicz W. Holocene palaeoecological changes recorded in mollusc-bearing cave sediments, the Cave above the Słupska Gate (southern Poland). Geol Acta. 2016 Sep 1;14(3):283–98.

19. Wojenka M, Wilczyński J, Sobieraj D. Archaeological excavations in the Żarska Cave, Krzeszowice commune, Kraków district, in 2011. Prądnik Pr i Mater Muz im Prof Władysława Szafera. 2012;22:77–97.

20. Stefaniak K, Muzolf B, Mirosław-Grabowska J, Socha P. Studies in the caves of the Zegarowe Rock. In: Stefaniak K, Tyc A, Socha P, editors. Karst of the Częstochowa Upland and of the Eastern Sudetes – palaeoenvironments and protection. Sosnowiec – Wrocław: Faculty of Earth Sciences, University of Silesia – Zoological Institute, University of Wrocław; 2009. p. 255–71.

21. Krajcarz MT. Geology, archaeology and paleozoology of the Perspektywiczna Cave sediments – state of knowledge 2016. Around Perspekt Cave Newsl. 2017;2:2.

22. Kondracki J. Geografia regionalna Polski, third ed. Warszawa: : Wydawnictwo Naukowe PWN; 2013.

23. Grodzicki J, editor. Jaskinie Wyżyny Częstochowskiej, vol. 4. Jaskinie Pasma Smoleńsko-Niegowonickiego. Warszawa: Wyd. PTPNoZ; 2011.

24. Kowalski K. Jaskinie Polski. Vol. 1, Jaskinie Wyżyny Krakowsko-Częstochowskiej. Warszawa: Państwowe Muzeum Archeologiczne; 1951.

25. Gradziński R. Sedymentacja piasków formierskich na skrasowiałym podłożu środkowej części Jury Krakowsko-Częstochowskiej. Kras i Speleol. 1977;1(10):59–70.

26. Lewandowski J. Pokrywy fluwioperyglacjalne Wyżyny Częstochowskiej. Przegląd Geol. 1994;42(12):1009–113.

27. Lewandowski J. „Glacial oasis" in the Kraków-Częstochowa Upland – results of studies during the last fifty years. Przegląd Geol. 2011;59(11):732–8.

28. Kaziuk H, Bednarek J, Zapaśnik T. Szczegółowa Mapa Geologiczna Polski, sheet 913 – Ogrodzieniec. Wydawnictwa: Wydawnictwa Geologiczne; 1976.

29. Madeyska T. Clastic cave sediments in the Częstochowa Upland. In: Stefaniak K, Tyc A, Socha P, editors. Karst of the Częstochowa Upland and of the Eastern Sudetes – palaeoenvironments and protection Studies of the Faculty of Earth Sciences, University of Silesia No 56. Sosnowiec – Wrocław: Faculty of Earth Sciences, University of Silesia – Zoological Institute, University of Wrocław; 2009. p. 67–84.

30. Mirosław-Grabowska J, Cyrek K. Archaeology and stratigraphy of the Jasna Strzegowska Cave. In: Stefaniak K, Tyc A, Socha P, editors. Karst of the Częstochowa Upland and of the Eastern Sudetes – palaeoenvironments and

protection Studies of the Faculty of Earth Sciences, University of Silesia No 56. Sosnowiec – Wrocław: Faculty of Earth Sciences, University of Silesia – Zoological Institute, University of Wrocław; 2009. p. 273–82.

31. Mirosław-Grabowska J. Geological value of Biśnik Cave sediments. Acta Geol Pol. 2002;52(1):97–110.

32. Sudoł M, Krajcarz M. Wyniki badań sondażowych w Schronisku w Górze Smoleń II oraz Schronisku w Smoleniu III, województwo śląskie. In: Glanc-Zagaja G, editor. Badania Archeologiczne na Górnym Śląsku i ziemiach pogranicznych w latach 2011-2012. Katowice: Śląskie Centrum Dziedzictwa Kulturowego w Katowicach; 2014. p. 13–21.

33. Sudoł M, Bokiniec E, Krajcarz M, Krajcarz M, Trojan A, Grafka O. Human activity traces from Shelter in Smoleń III (central part of Kraków-Częstochowa Upland) from the last centuries of antiquity. Przegląd Archeol. 2015;63:177–93.

34. Sudoł M, Cyrek K, Krajcarz M, Krajcarz M. Around the Biśnik Cave – the area of human penetration during Palaeolithic. Anthropol (Czech Republic). 2016;54(1):49–68.

35. Krajcarz M, Makowiecki D, Krajcarz MT, Masłowska A, Baca M, Panagiotopoulou H, et al. On the trail of the oldest domestic cat in Poland. An insight from morphometry, ancient DNA and radiocarbon dating. Int J Osteoarchaeol. 2016 Sep 1;26(5):912–9.

36. Madeyska-Niklewska T. Methods used in researches on the upper pleistocene sediments of the Cracov Upland caves. Światowit. 1971;32:5–25.

37. Antoine P, Rousseau DD, Degeai JP, Moine O, Lagroix F, Kreutzer S, et al. High-resolution record of the environmental response to climatic variations

during the Last Interglacial-Glacial cycle in Central Europe: The loess-palaeosol sequence of Dolní Věstonice (Czech Republic). Quat Sci Rev. 2013 May 1;67:17–38.

38. Krajcarz MT, Madeyska T. Application of the weathering parameters of bones to stratigraphical interpretation of the sediments from two caves (Deszczowa Cave and Nietoperzowa Cave, Kraków-Czestochowa Upland, Poland). Stud Quat. 2010;27:43–54.

39. Krajcarz MT. Alteration of the metal content in animal bones after 2.5-year experimental exposure to sediments. Archaeol Anthropol Sci. 2019 Jan 1;11(1):361–72.

40. Myślińska E. Laboratoryjne badania gruntów. Warszawa: PWN; 2006.

41. Brock F, Higham T, Ditchfield P, Ramsey CB. Current pretreatment methods for AMS radiocarbon dating at the Oxford Radiocarbon Accelerator Unit (ORAU). Radiocarbon. 2010;52(1):103–12.

42. Goslar T, Czernik J, Goslar E. Low-energy 14C AMS in Poznań Radiocarbon Laboratory, Poland. In: Nuclear Instruments and Methods in Physics Research, Section B: Beam Interactions with Materials and Atoms. 2004. p. 5–11.

43. Deniro MJ. Postmortem preservation and alteration of in vivo bone collagen isotope ratios in relation to palaeodietary reconstruction. Nature. 1985;317(6040):806–9.

44. Ambrose SH. Preparation and characterization of bone and tooth collagen for isotopic analysis. J Archaeol Sci. 1990;17(4):431–51.

45. Bronk Ramsey C, Lee S. Recent and Planned Developments of the Program OxCal. Radiocarbon. 2013;55(2–3).

46. Reimer PJ, Baillie MGL, Bard E, Bayliss A, Beck JW, Blackwell PG, et al. IntCal09 and Marine09 radiocarbon age calibration curves, 0-50,000 years CAL BP. Radiocarbon. 2009;51(4):1111–50.

47. Krajcarz MT, Krajcarz M, Ginter B, Goslar T, Wojtal P. Towards a chronology of the Jerzmanowician - a new series of radiocarbon dates from Nietoperzowa Cave (Poland). Archaeometry. 2018 Apr 1;60(2):383–401.

48. Bronk Ramsey C. Radiocarbon Calibration and Analysis of Stratigraphy: The OxCal Program. Radiocarbon. 1995;37(2):425–30.

49. Bronk Ramsey C. Bayesian Analysis of Radiocarbon Dates. Radiocarbon. 2009;51(1):337–60.

50. Moska P, Jary Z, Adamiec G, Bluszcz A. OSL chronostratigraphy of a loess-palaeosol sequence in Zlota using quartz and polymineral fine grains. Radiat Meas. 2015 Oct 1;81:23–31.

51. Thiel C, Buylaert J-P, Murray AS, Terhorst B, Tsukamoto S, Frechen M, et al. Investigating the chronostratigraphy of prominent palaeosols in Lower Austria using post-IR IRSL dating. E&G Quat Sci J. 2011 Jun 6;60(1):137–52.

52. Ložek V. Quartärmollusken der Tschechoslowakei. Praha: Tschechoslowakische Akademie der Wissenschaften; 1964.

53. Ložek V. Mollusca Analysis. In: Berglund B, editor. Handbook of Holocene Palaeoecology and Palaeohydrology. Caldwell, New Jersey: The Blackburn Press; 1986. p. 729–40.

54. Ložek V. Palaeoecology of Quaternary Mollusca. Antropozoikum. 2000;24:35–59.

55. Alexandrowicz S, Alexandrowicz W. Analiza malakologiczna, metody badań i interpretacji. Rozpr Wydz Przyr PAU. 2011;3:5–302.

56. Kerney M, Cameron R, Jungbluth J. Die Landschnecken Nord- und Mitteleuropas. Hamburg-Berlin: Veralg P. Parey; 1983.

57. Wiktor A. Ślimaki lądowe Polski. Olsztyn: Wydawnictwo Mantis; 2004.

58. Welter-Schultes F. European non-marine molluscs, a guide for species identification. Goettingen: Planet Poster Editions; 2012.

59. Pucek Z, editor. Klucz do oznaczania ssaków Polski. Warszawa: Państwowe Wydawnictwo Naukowe; 1984.

60. Niethammer J, Krapp F, editors. Handbuch der Säugetiere Europas. Band 2/1, Rodentia II : (Cricetidae, Arvicolidae, Zapodidae, Spalacidae, Hystricidae, Capromyide). Wiesbaden: Akademische Verlagsgesellschaft; 1982.

61. Nadachowski A. Late Quaternary rodents of Poland with special reference to morphotype dentition analysis of voles. Warszawa-Kraków: Państwowe Wydawn. Nauk; 1982. 108 p.

62. Nadachowski A. Taxonomic value of anteroconid measurements of M1 in common and field voles. Acta Theriol (Warsz). 1984;29:123–7.

63. Wilson D, Reeder D, editors. Mammal Species of the World A Taxonomic and Geographic Reference. 3rd editio. Baltimore, Maryland: Johns Hopkins University Press; 2005. 142 p.

64. Tesakov A, Lebedev V, Bannikova A, Abramson N. Clethrionomys Tilesius, 1850 is the valid generic name for red-backed voles and Myodes Pallas, 1811 is a junior synonym of Lemmus Link, 1795. Russ J Theriol. 2010;9:83–6.

65. Abramson N, Lissovsky A. Subfamily Arvicolinae. In: Pavlinov I, Lissovsky A, editors. The Mammals of Russia: A taxonomic and Geographic Reference (Archive of the Zoological Museum of MSU Vol 52). Moscow: KMK Scientific Press; 2012. p. 220–76.

66. Lyman R. Vertebrate Taphonomy. Cambridge: Cambridge University Press; 1994. 1–552 p.

67. Lyman R. Quantitative Paleozoology. Cambridge: Cambridge University Press; 2008. 1–374 p.

68. Menu H, Popelard J-B. Utilisation des caractères dentaires pour la détermination des Vespertilioninés de l'Ouest Européen. Le Rhinolophe. 1987;4:1–88.

69. Dodelin B. Identification des Chiroptères de France á partir de restes osseux. Fédération française de spéléologie; 2002. 48 p.

70. Lanza B. Fauna d'Italia, volume 47: Mammalia V, Chiroptera. Milano: Ed. Calderini; 2012. 786 p.

71. Horn HS. Measurement of "Overlap" in Comparative Ecological Studies. Am Nat. 1966 Sep;100(914):419–24.

72. Hammer DAT, Ryan PD, Hammer Ø, Harper DAT. PAST: Paleontological Statistics Software Package for Education and Data Analysis. Vol. 4, Palaeontologia Electronica. 2001.

73. Andrews P. Taphonomic effects of faunal impoverishment and faunal mixing. Palaeogeogr Palaeoclimatol Palaeoecol. 2006 Nov 14;241(3–4):572–89.

74. Cuenca-Bescós G, Straus LG, González Morales MR, García Pimienta JC. The reconstruction of past environments through small mammals: from the

Mousterian to the Bronze Age in El Mirón Cave (Cantabria, Spain). J Archaeol Sci. 2009;36(4):947–55.

75. López-García JM, Berto C, Colamussi V, Valle CD, Vetro D Lo, Luzi E, et al. Palaeoenvironmental and palaeoclimatic reconstruction of the latest Pleistocene-Holocene sequence from Grotta del Romito (Calabria, southern Italy) using the small-mammal assemblages. Palaeogeogr Palaeoclimatol Palaeoecol. 2014 Sep 1;409:169–79.

76. Berto C, Boscato P, Boschin F, Luzi E, Ronchitelli A. Paleoenvironmental and paleoclimatic context during the Upper Palaeolithic (late Upper Pleistocene) in the Italian Peninsula. The small mammal record from Grotta Paglicci (Rignano Garganico, Foggia, Southern Italy). Quat Sci Rev. 2017 Jul 15;168:30–41.

77. Mitchell-Jones A, Amori G, Bogdanowicz W, Krystufek B, Reijnders P, Spitzenberger F, et al. The atlas of European mammals. London: T & AD Poyser; 1999. 496 p.

78. Bertolino S, Amori G, Henttonen H, Zagorodnyuk I, Zima J, Juškaitis R, et al. Eliomys quercinus. IUCN Red List Threat Species. 2008;e.T7618A12.

79. Amori G, Hutterer R, Kryštufek B, Yigit N, Mitsain G, Palomo L. Apodemus flavicollis. IUCN Red List Threat Species. 2016;e.T1892A11.

80. Amori G, Hutterer R, Kryštufek B, Yigit N, Mitsain G, Muñoz L, et al. Glis glis. IUCN Red List Threat Species. 2016;e.T39316A1.

81. Amori G, Hutterer R, Yigit N, Mitsain G, Kryštufek B, Vohralík V, et al. Microtus subterraneus. IUCN Red List Threat Species. 2016;e.T13489A1.

82. Aplin K, Lunde D, Batsaikhan N, Kryštufek B, Meinig H, Henttonen H. Micromys minutus. IUCN Red List Threat Species. 2016;e.T13373A1.

83. Batsaikhan N, Henttonen H, Meinig H, Shenbrot G, Bukhnikashvili A, Hutterer R, et al. Arvicola amphibius. IUCN Red List Threat Species. 2016;e.T2149A11.

84. Cserkész T, Kennerley R. Sicista subtilis (errata version published in 2018). IUCN Red List Threat Species. 2017;e.T9193444.

85. Henttonen H. Lemmus lemmus. IUCN Red List Threat Species. 2016;e.T11481A5.

86. Hutterer R, Kryštufek B, Yigit N, Mitsain G, Palomo L, Henttonen H, et al. Myodes glareolus. IUCN Red List Threat Species. 2016;e.T4973A11.

87. Hutterer R, Kryštufek B, Yigit N, Mitsain G, Meinig H, Juškaitis R. Muscardinus avellanarius. IUCN Red List Threat Species. 2016;e.T13992A1.

88. Kryštufek B, Vohralík V, Meinig H, Zagorodnyuk I. Cricetus cricetus. IUCN Red List Threat Species. 2016;e.T5529A11.

89. Kryštufek B, Vohralík V, Zima J, Zagorodnyuk I. Microtus agrestis. IUCN Red List hreatened Species. 2016;e.T13426A1.

90. Linzey A, Shar S, Lkhagvasuren D, Juškaitis R, Sheftel B, Meinig H, et al. Microtus oeconomus. IUCN Red List Threat Species. 2016;e.T13451A1.

91. Meinig H, Zagorodnyuk I, Henttonen H, Zima J, Coroiu I. Sicista betulina. IUCN Red List Threat Species. 2016;e.T20184A1.

92. Shar S, Lkhagvasuren D, Bertolino S, Henttonen H, Kryštufek B, Meinig H. Sciurus vulgaris. IUCN Red List Threat Species. 2016;e.T20025A1.

93. Schlitter D, van der Straeten E, Amori G, Hutterer R, Kryštufek B, Yigit N, et al. Apodemus sylvaticus. IUCN Red List Threat Species. 2016;e.T1904A11.

94. Tsytsulina K, Formozov N, Sheftel B. Dicrostonyx torquatus. IUCN Red List

Threat Species. 2016;e.T6568A11.

95. Yigit N, Hutterer R, Kryštufek B, Amori G. Microtus arvalis. IUCN Red List Threat Species. 2016;e.T13488A2.

96. Batsaikhan N, Tsytsulina K, Formozov N, Sheftel B. Microtus gregalis. IUCN Red List Threat Species. 2016;e.T13431A1.

97. Entwistle A, Racey P, Speakman J. Habitat exploitation by a gleaning bat, Plecotus auritus. Philos Trans R Soc B Biol Sci. 1996;351(1342):921–31.

98. Dietz M, Pir B. Distribution and habitat selection of Myotis bechsteinii in Luxembourg: implications for forest management and conservation. Folia Zool. 2009;58(3):327–40.

99. Zeale MRK, Davidson-Watts I, Jones G. Home range use and habitat selection by barbastelle bats (Barbastella barbastellus): implications for conservation. J Mammal. 2012 Sep 14;93(4):1110–8.

100. Parson N, Jones G. Dispersion and habitat use by Myotis daubentonii and Myotis nattereri during the swarming season: implications for conservation. Anim Conserv. 2003;6:283–90.

101. Van De Sijpe M, Holsbeek L. Hunting strategy and tympanate moth predation by the pond bat (Myotis dasycneme). Lutra. 2007;50(2):91–106.

102. Baca M, Popović D, Panagiotopoulou H, Marciszak A, Krajcarz M, Krajcarz MT, et al. Human-mediated dispersal of cats in the Neolithic Central Europe. Heredity (Edinb). 2018;121(6):557–63.

103. Alexandrowicz SSW. Malacofauna of Holocene cave sediments of the Cracow Upland (Southern Poland). Folia Quat. 2000;71:85–112.

104. Alexandrowicz W. Molluscan assemblages from cave and slope sediments of the Częstochowa Upland (Poland). Folia Quat. 2000;71:113–37.

105. Alexandrowicz WP. Malacological sequence from profile of calcareous tufa in Groń (Podhale Basin, southern Poland) as an indicator of the Late Glacial/Holocene boundary. Quat Int. 2013 Apr 19;293:196–206.

106. Alexandrowicz WP, Szymanek M, Rybska E. Changes to the environment of intramontane basins in the light of malacological research of calcareous tufa: Podhale basin (Carpathians, Southern Poland). Quat Int. 2014;353(1):250–65.

107. Alexandrowicz WP, Szymanek M, Rybska E. Molluscan assemblages from Holocene calcareous tufa and their significance for palaeoenvironmental reconstructions. A study in the Pieniny Mountains (Carpathians, Southern Poland). Carpathian J Earth Environ Sci. 2016;11(1):37–54.

108. Schileyko A. Land mollusc suborder Pupillina of USSR fauna (Gastropoda, Pulmonata, Geophila). Fauna of the USSR, New Series, No. 130. Leningrad: Nauka; 1984.

109. White D, Preece RC, Shchetnikov AA, Parfitt SA, Dlussky KG. A Holocene molluscan succession from floodplain sediments of the upper Lena River (Lake Baikal region), Siberia. Quat Sci Rev. 2008 May;27(9–10):962–87.

110. Horsák M, Chytrý M, Pokryszko BM, Danihelka J, Ermakov N, Hájek M, et al. Habitats of relict terrestrial snails in southern Siberia: Lessons for the reconstruction of palaeoenvironments of full-glacial Europe. J Biogeogr. 2010 Aug;37(8):1450–62.

111. Horsák M, Chytrý M, Hájková P, Hájek M, Danihelka J, Horsáková V, et al. European glacial relict snails and plants: Environmental context of their modern

refugial occurrence in southern Siberia. Boreas. 2015 Oct 1;44(4):638–57.

112. Chmielewski W. Stanowisko paleolityczne w Dziadowej Skale koło Skarżyc w pow. zawierciańskim. Pr i Mater Muz Archeol i Etnogr w Łodzi, Ser Archeol. 1958;3:5–48.

113. Bosák P, Głazek J, Horáček I, Szynkiewicz A. New locality of Early Pleistocene vertebrates – Żabia Cave at Podlesice, Central Poland. Acta Geol Pol. 1982;32:217–26.

114. Różycki S. Plejstocen Polski Środkowej. Warszawa: PWN; 1972.

115. Madeyska T. The stratigraphy of Palaeolithic sites of the Cracow Upland. Acta Geol Pol. 1982;32:227–42.

116. Krajcarz M, Sudoł M, Krajcarz M, Cyrek K. Stanowisko późnoczwartorzędowych osadów jaskiniowych – Schronisko nad Jaskinią Zegar w Skałach Zegarowych (Wyżyna Częstochowska). Przegląd Geol. 2012;60(10):546–53.

117. Włodarz B. Zabytkowa kopalnia górnictwa kruszcowego w Olkuszu – perspektywy uruchomienia. Górnictwo i Geoinżynieria. 2006;30(4):131–41.

118. Niemann D. Secrets of a badger’s toilet. Guard. 2014;Sep 18;05.

119. Buesching CD, Newman C, Service K, Macdonald DW, Riordan P. Latrine marking patterns of badgers (Meles meles) with respect to population density and range size. Ecosphere. 2016 May 1;7(5):e01328.

120. Starkel L, Michczyńska D, Krąpiec M, Margielewski W, Nalepka D, Pazdur A. Progress in the Holocene chrono-climatostratigraphy of Polish territory. Geochronometria. 2013;40(1):1–21.

121. Cohen KM, Finney SC, Gibbard PL, Fan J-X. The ICS International Chronostratigraphic Chart. Episodes. 36(3):199–204.

122. Stworzewicz E. Kopalna fauna ślimaków (Gastropoda) ze schroniska nad Jaskinią Niedostępną w okolicach Ojcowa. Acta Zool Cracoviensia. 1973;18:301–9.

123. Stworzewicz E. Fauna mięczaków jaskiń i schronisk Doliny Sąspowskiej. In: Chmielewski W, editor. Jaskinie Doliny Sąspowskiej Tło przyrodnicze osadnictwa pradziejowego. Warszawa: Wyd. Uniwersytetu Warszawskiego; 1988. p. 39–45.

124. Alexandrowicz SW, Drobniewicz B, Ginter B, Kozłowski JK, Madeyska T, Nadachowski A, et al. Excavations in the Zawalona cave at Mnikow (Cracow upland, southern Poland). Folia Quat. 1992;63:43–76.

125. Alexandrowicz W, Stworzewicz E. Snails (Gastropoda). In: Valde-Nowak P, Nadachowski A, Madeyska T, editors. Obłazowa cave Human activity, stratigraphy and palaeoenvironment. Cracow: Institute of Archaeology and Ethnology PAS; 2003. p. 91–4.

126. Ložek V. Molluscan and Vertebrate Successions from the Veľká Drienčanská Cave. Malacol Bohemoslov. 2012;11:39–44.

127. Svoboda J, Horáček I, Ložek V, Svobodová H, Šilar J. The Pekárna Cave. Magdalenian stratigraphy, environment, and the termination of the loess formation in Moravian Karst. Sb Geol Ved - Antropozoikum. 2000;24(24):61–79.

128. Bocheński Z, Gintner B, Kozłowski J, Mook W, Muszyński M, Nadachowski A, et al. Badania osadów schronisk podskalnych w Zalasie koło Krakowa. Folia

Quat. 1985;56:3–56.

129. Alexandrowicz S. Malacofauna from a rock-shelter in Ruskie Skały Tors (Będkowska Valley, Cracow Upland). Folia Quat. 1992;63:27–34.

130. Alexandrowicz W. Malacofauna of Quaternary deposits and environmental changes of the Podhale Basin during the Late Glacial and Holocene. Folia Quat. 1997;68:7–132.

131. Alexandrowicz WP. Late Vistulian and Holocene mollusc assemblages from calcareous tufa at the Ostrysz Hill (Podhale Basin, S Poland). Folia Malacol. 2001 Sep 10;9(3):159–69.

132. Alexandrowicz W, Rudzka D. Molluscan communities from cave and slope deposits of the limestone rocky hills in the eastern part of Podhale Basin (Southern Poland). Folia Malacol. 2006;14:191–201.

133. Alexandrowicz S, Nadachowski A, Rydlewski J, Valde-Nowak P, Wołoszyn B. Subfossil fauna from cave sediments in the Sobczański Gully (Pieniny Mts. Poland). Folia Quat. 1985;56:57–78.

134. Juřičková L, Horsák M, Horáčková J, Abraham V, Ložek V. Patterns of land-snail succession in Central Europe over the last 15,000 years: Main changes along environmental, spatial and temporal gradients. Quat Sci Rev. 2014 Jun 1;93:155–66.

135. Andrews P. Owls, caves, and fossils : predation, preservation, and accumulation of small mammal bones in caves, with an analysis of the Pleistocene cave faunas from Westbury-sub-Mendip, Somerset, UK. University of Chicago Press; 1990. 231 p.

136. Kowalski K. Plejstoceńskie gryzonie Jaskini Nietoperzowej w Polsce. Folia

Quat. 1961;5:1–22.

137. Nadachowski A. Origin and history of the present rodent fauna in Poland based on fossil evidence. Acta Theriol (Warsz). 1989;34(2):37–53.

138. Nowak J, Grzywiński W. Zimowe spisy nietoperzy na Wyżynie Krakowskiej w latach 2013-2017 na tle historii badań. Prądnik Pr i Mater Muz im Prof Władysława Szafera. 2017;(27):93–118.

139. Madeyska T. Vegetation in Poland at the close of last glaciation. Stud Geol Pol. 1998;113:137–80.

140. Alexandrowicz W. Molluscan assemblages of Late Glacial and Holocene calcareous tufas in Southern Poland. Folia Quat. 2004;75:1–309.

141. Alexandrowicz WP. Molluscan assemblages in Late Holocene tufa cones in the Pieniny mountains (southern Poland). Geol Q. 2014;58(2):269–80.

142. Alexandrowicz SW, Alexandrowicz Z. Recurrent Holocene landslides: a case study of the Krynica landslide in the Polish Carpathians. The Holocene. 1999 Jan 27;9(1):91–9.

143. Dehm R. Die landschnecke Discus ruderatus im Postglazial Süddeutschlands. Mitteilungen der Bayer Staatssammlung für Paläontologie und Hist Geol. 1967;(7):135–55.

144. Preece RC, Day SP. Comparison of Post-Glacial Molluscan and Vegetational Successions from a Radiocarbon-Dated Tufa Sequence in Oxfordshire. J Biogeogr. 1994 Sep;21(5):463.

145. Füköh L. History of the Hungarian Holocene mollusc fauna. GeoJournal. 1995;36(3):255–9.

146. Preece RC, Bridgland DR. Holywell Coombe, Folkestone: A 13,000 year history of an English Chalkland Valley. Quat Sci Rev. 1999;18(8–9):1075–125.

147. Gedda B. Environmental and climatic aspects of the Early and Mid Holocene calcareous tufa and land mollusc fauna in southern Sweden. Doctoral thesis, Lund University; 2001.

148. Meyrick RA. Holocene molluscan faunal history and environmental change at Kloster Mühle, Rheinland-Pfalz, western Germany. J Quat Sci. 2003 Feb;18(2):121–32.

149. Limondin-Lozouet N, Preece RC. Molluscan successions from the Holocene tufa of St Germain-le-Vasson, Normandy (France) and their biogeographical significance. J Quat Sci. 2004 Jan;19(1):55–71.

150. Limondin-Lozouet N. Successions malacologiques à la charnière Glaciaire/Interglaciaire: du modèle tardiglaciaire‐ Holocène aux transitions du Pleistocène. Quaternaire. 2011 Sep 1;22(3):211–20.

151. Nadachowski A. Fauna kopalna płazów (Amphibia), gadów (Reptilia) i ssaków (Mammalia) w osadach jaskiń i schronisk Doliny Sąspowskiej. In: Chmielewski W, editor. Jaskinie Doliny Sąspowskiej Tło przyrodnicze osadnictwa pradziejowego. Warszawa: Wyd. Uniwersytetu Warszawskiego; 1988. p. 19–38.

152. Fuhrmann R. Die spätweichselglaziale und holozäne Molluskenfauna Mittel – und Westsachsens. Freib Forschungshefte, R C. 1973;278:1–121.

153. Dehm R. Die landschnecke Discus perspectivus im Postglazial Südbayerns. Mitteilungen der Bayer Staatssammlung für Paläontologie und Hist Geol. 1987;27:21–30.

154. Alexandrowicz W, Rybska E. Environmental changes of intramontane basins

derived from malacological analysis of profile of calcareous tufa in Niedzica (Podhale Basin, Southern Poland). Carpathian J Earth Environ Sci. 2013;8(4):13–26.

155. Starkel L. Paleogeografia holocenu. Warszawa: PWN; 1977.

156. Ralska-Jasiewiczowa M. Ewolucja szaty roślinnej. In: Starkel L, editor. Geografia Polski, środowisko przyrodnicze. Warszawa: Wydawnictwo Naukowe PWN; 1991. p. 106–27.

157. Alexandrowicz S. Malacofauna of the Holocene calcareous sediments of the Cracow Upland. Acta Geol Pol. 1983;33(3):117–58.

158. Riedel A. Katalog fauny polski. Ślimaki lądowe. Warszawa: PWN; 1988.

159. Latałowa M, Ralska-Jasiewiczowa, M Miotk-Szpiganowicz, G Zachowicz J, Nalepka D. Fagus sylvatica L. – Beech. In: Ralska-Jasiewiczowa M, Latałowa M, Wasylikowa K, Tobolski K, Madeyska E, Wright HJ, et al., editors. Late Glacial and Holocene history of vegetation in Poland based on isopollen maps. Kraków: W. Szafer Institute of Botany, Polish Academy of Sciences; 2004. p. 95–104.

160. Cyrek K, editor. Jaskinia Biśnik. Rekonstrukcja zasiedlenia jaskini na tle zmian środowiska przyrodniczego. Toruń: Wyd. UMK; 2002.

161. Pelisiak A. Ze studiów nad wykorzystywaniem surowców krzemiennych ze środkowej części Wyżyny Krakowsko-Częstochowskiej w późnym neolicie w strefie karpackiej. Neolityczne pracownie w Strzegowej (Strzegowa, stan. 42). Acta Archaeol Carpathica. 2003;38:27–69.

162. Pelisiak A. The Exploitation and Distribution of Flints From the Central Part of Polish Jura in the Late Neolithic Times. Analecta Archaeol Ressoviensia.

2006;1:73–86.

163. Sudoł M, Adamczak K, Krajcarz M, Krajcarz M. Ślad osadnictwa z wczesnej epoki brązu w Schronisku w Udorzu II (Udórz, gm. Żarnowiec, woj. śląskie). Acta Univ Nicolai Copernici, Archeol. 2013;33:53–68.

164. Woźniak Z. Jura Ojcowska w okresie lateńskim (przedrzymskim). In: Lech J, Partyka J, editors. Jura Ojcowska w pradziejach i w początkach państwa polskiego. Ojców: Ojcowski Park Narodowy; 2006. p. 489–505.

165. Wagner T. Osadnictwo kultury przeworskiej na Wyżynie Krakowsko-Częstochowskiej u schyłku starożytności. B.Sc thesis, Jagiellonian University; 2008.

166. Bocheński Z, Młynarski M, Nadachowski, A Stworzewicz E, Wołoszyn B. Górnoholoceńska fauna z Jaskini Dużej Sowy (doniesienie wstępne). Przegląd Zool. 1983;27(4):437–55.

Appendix

Appendix S1: Lithological and geochemical parameters of sediments

Appendix S2: Mollusks – data on habitats, number of specimens, individuals, assemblages and diversity

Appendix S3: Rodents – data on habitats, number of specimens, individuals, assemblages and diversity

Appendix S4: Bats – data on number of specimens, individuals, assemblages and diversity

Figure 1 Click here to download Figure Figure 1.jpg

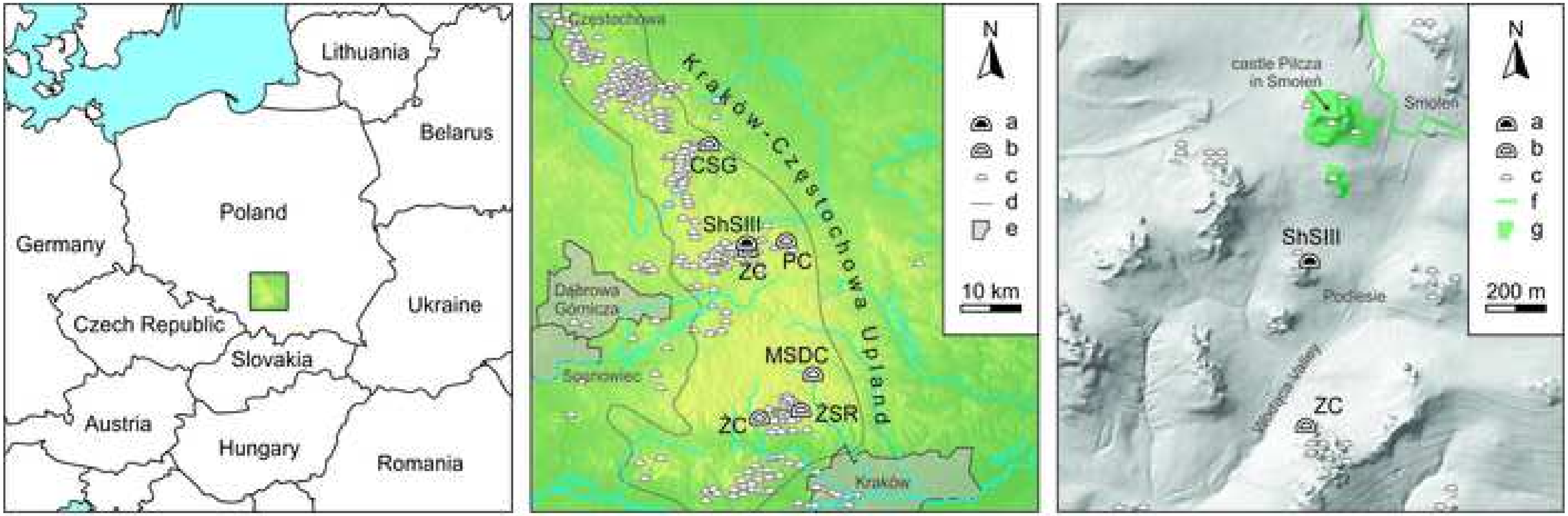

Figure 2

Click here to download Figure Figure 2.jpg

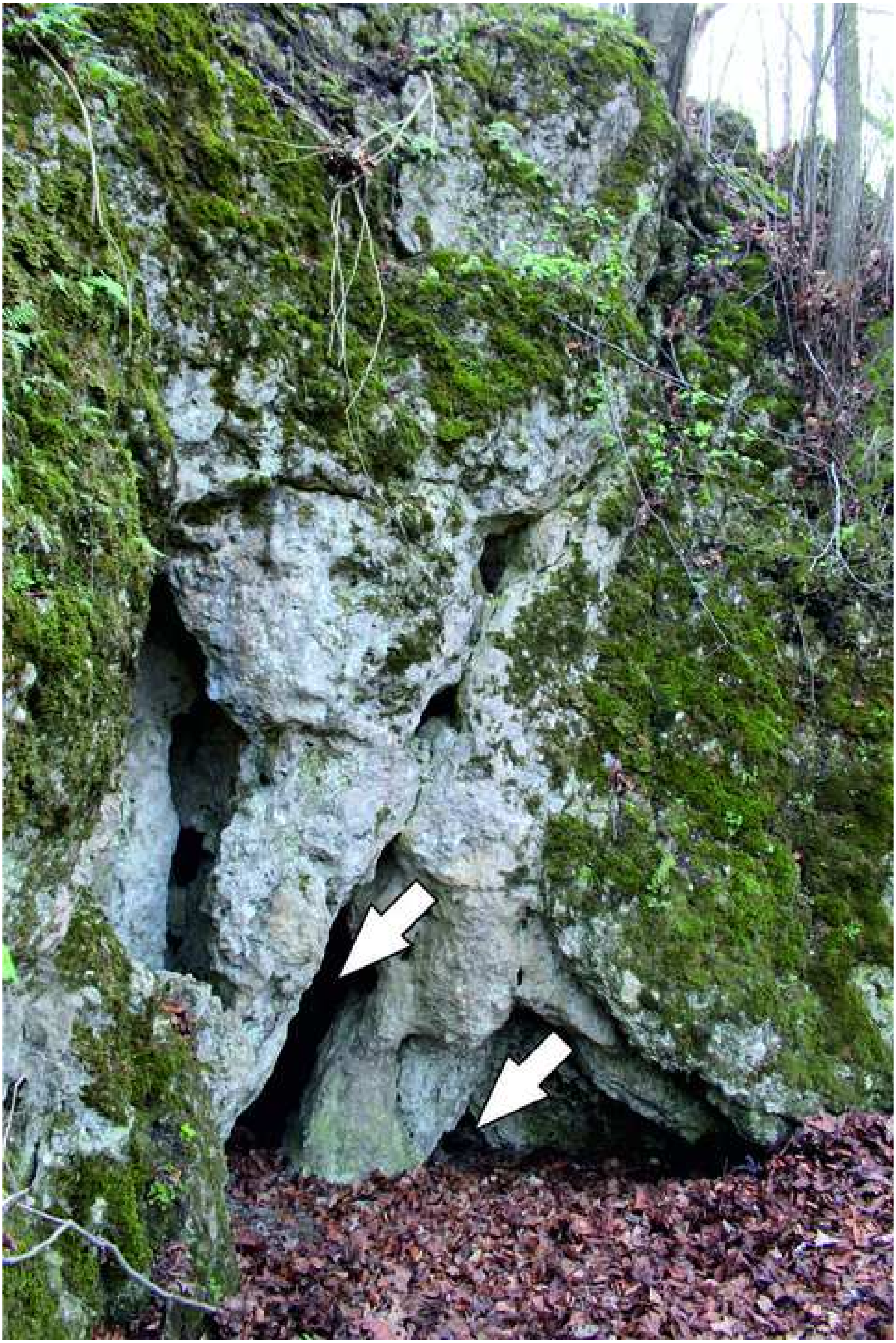

Figure 3 Click here to download Figure Figure 3.jpg

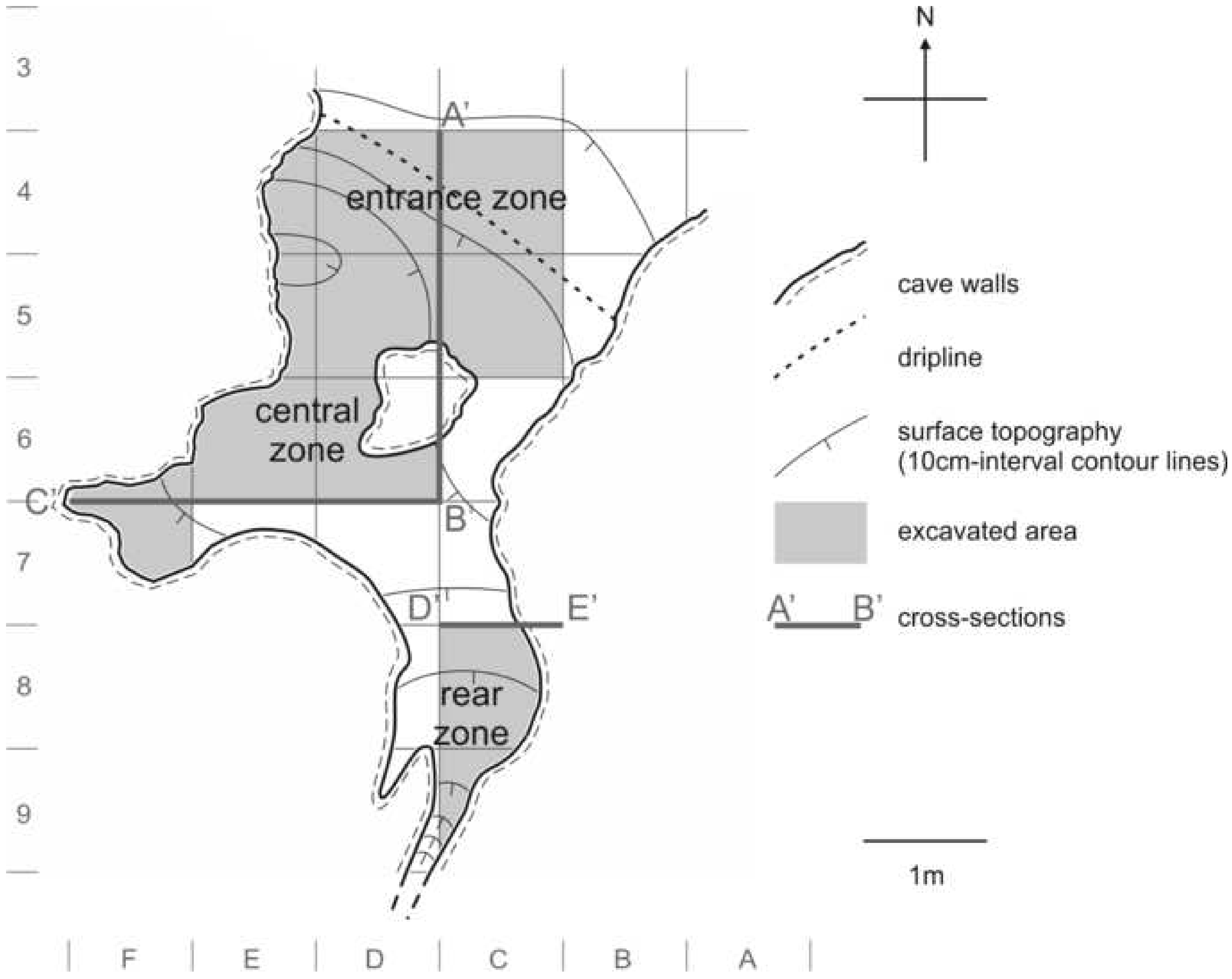

Figure 4

Click here to download Figure Figure 4.jpg

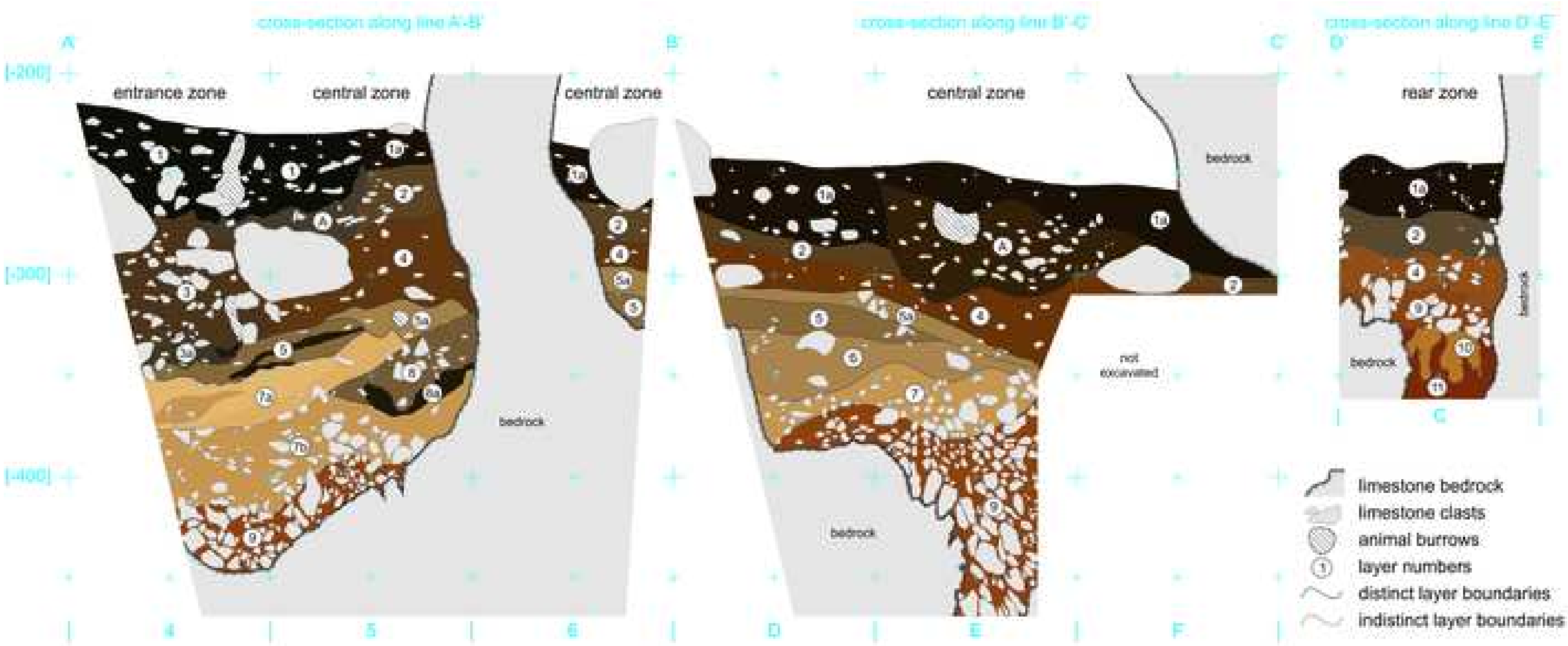

Figure 5

Click here to download Figure Figure 5.jpg

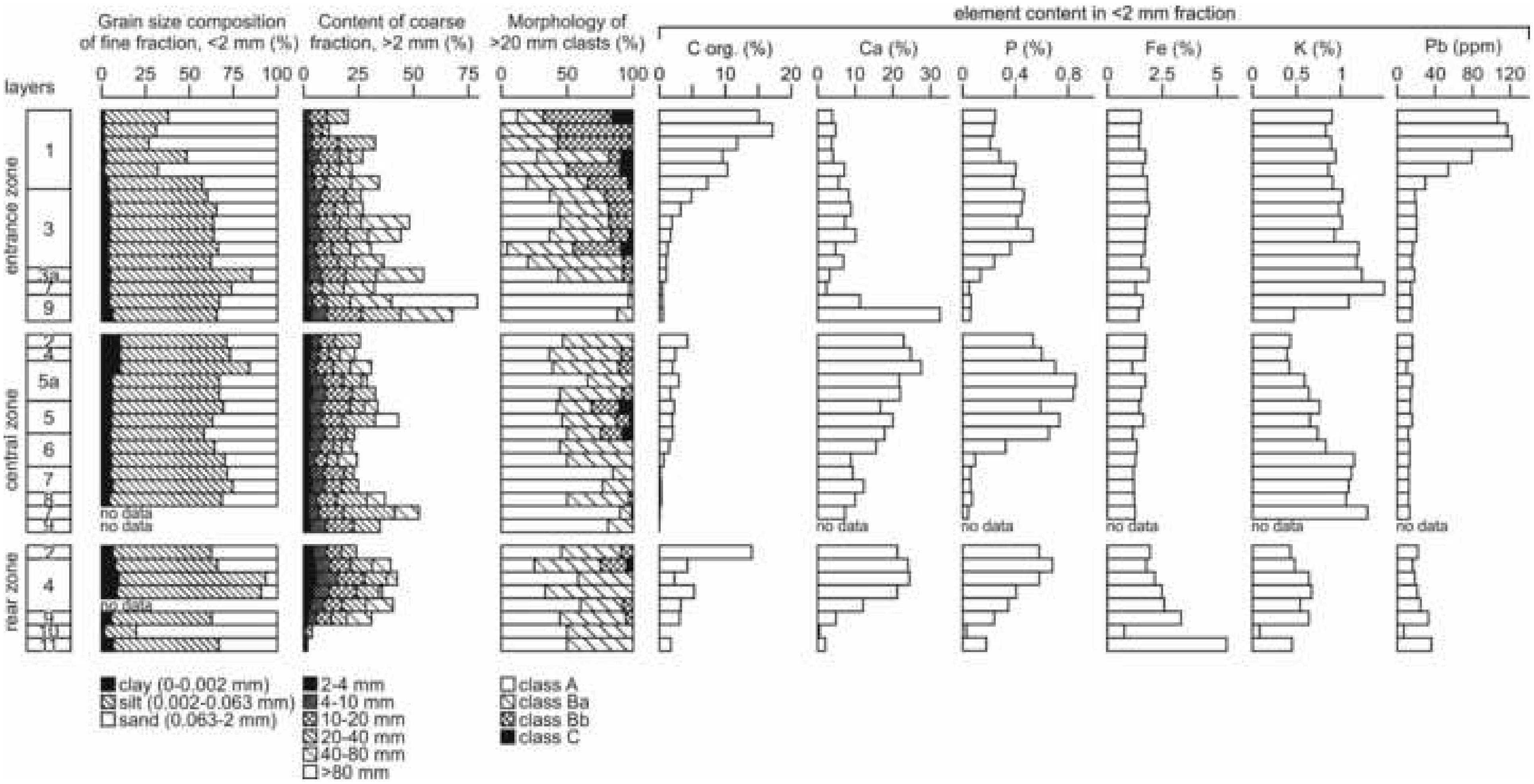

Figure 6

Click here to download Figure Figure 6.jpg

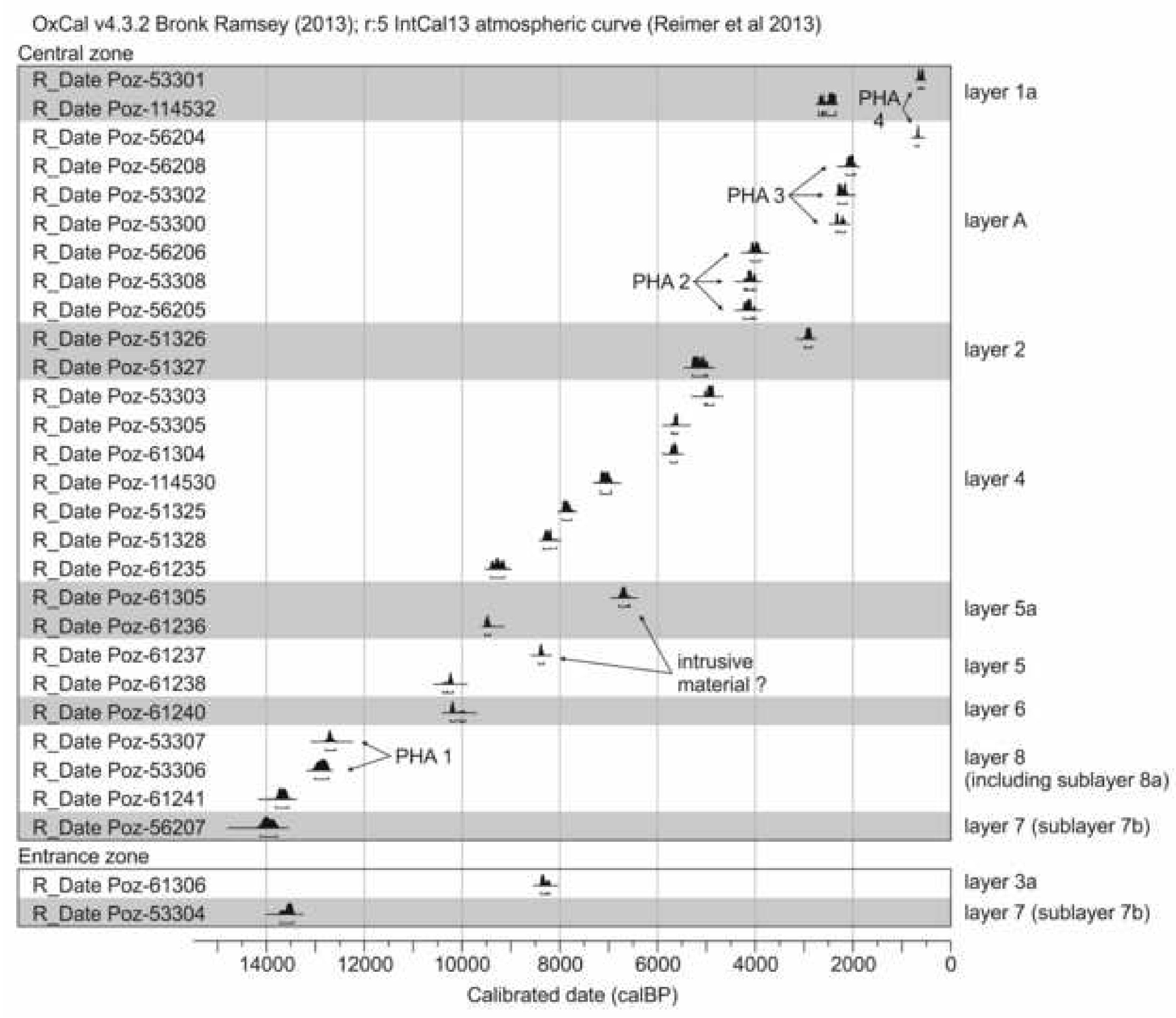

Figure 7

Click here to download Figure Figure 7.jpg

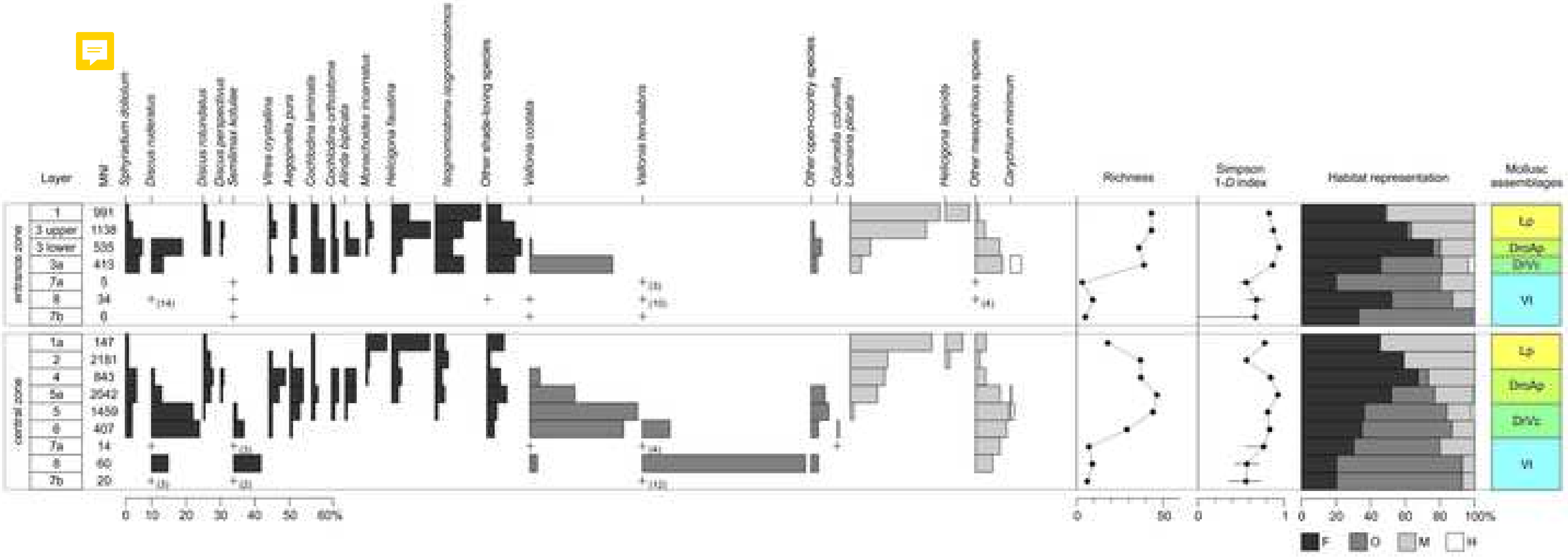

Figure 8

Click here to download Figure Figure 8.jpg

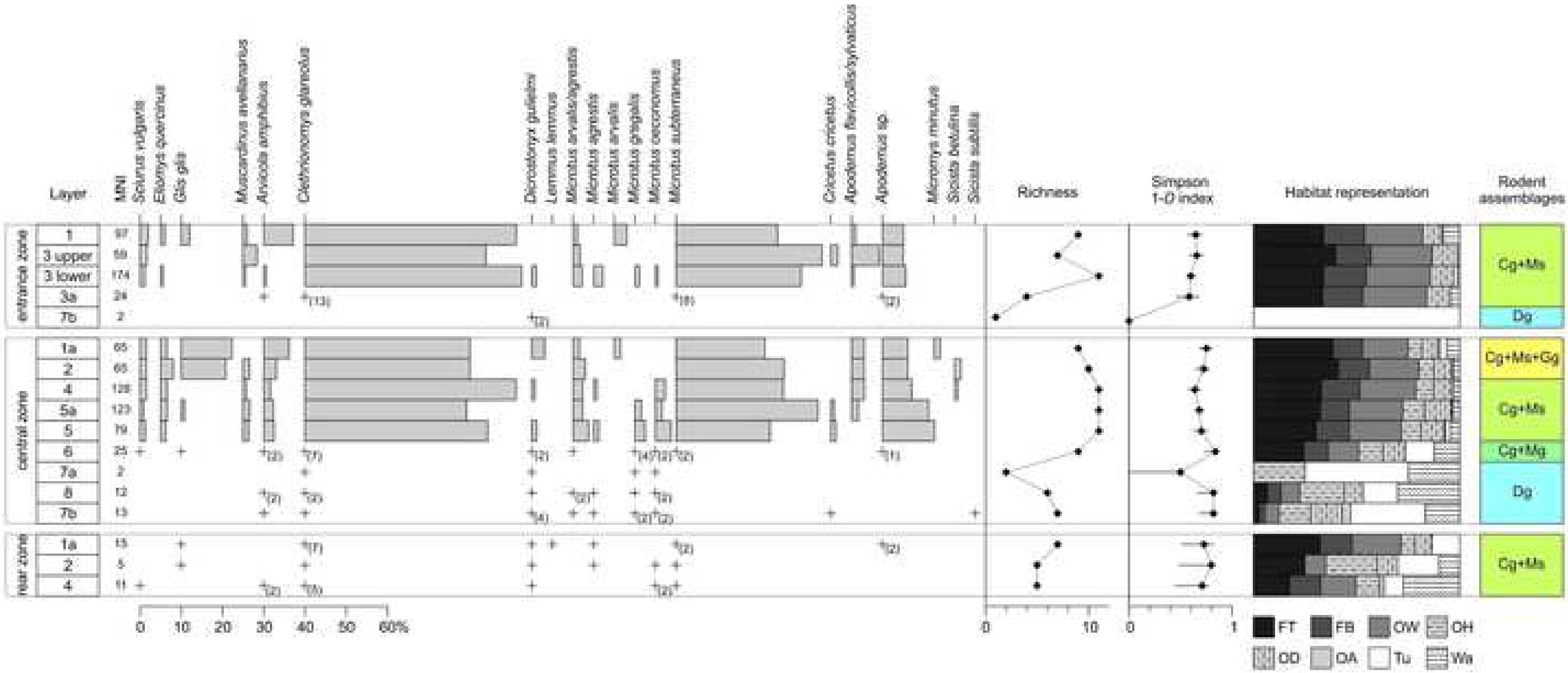

Figure 9

Click here to download Figure Figure 9.jpg

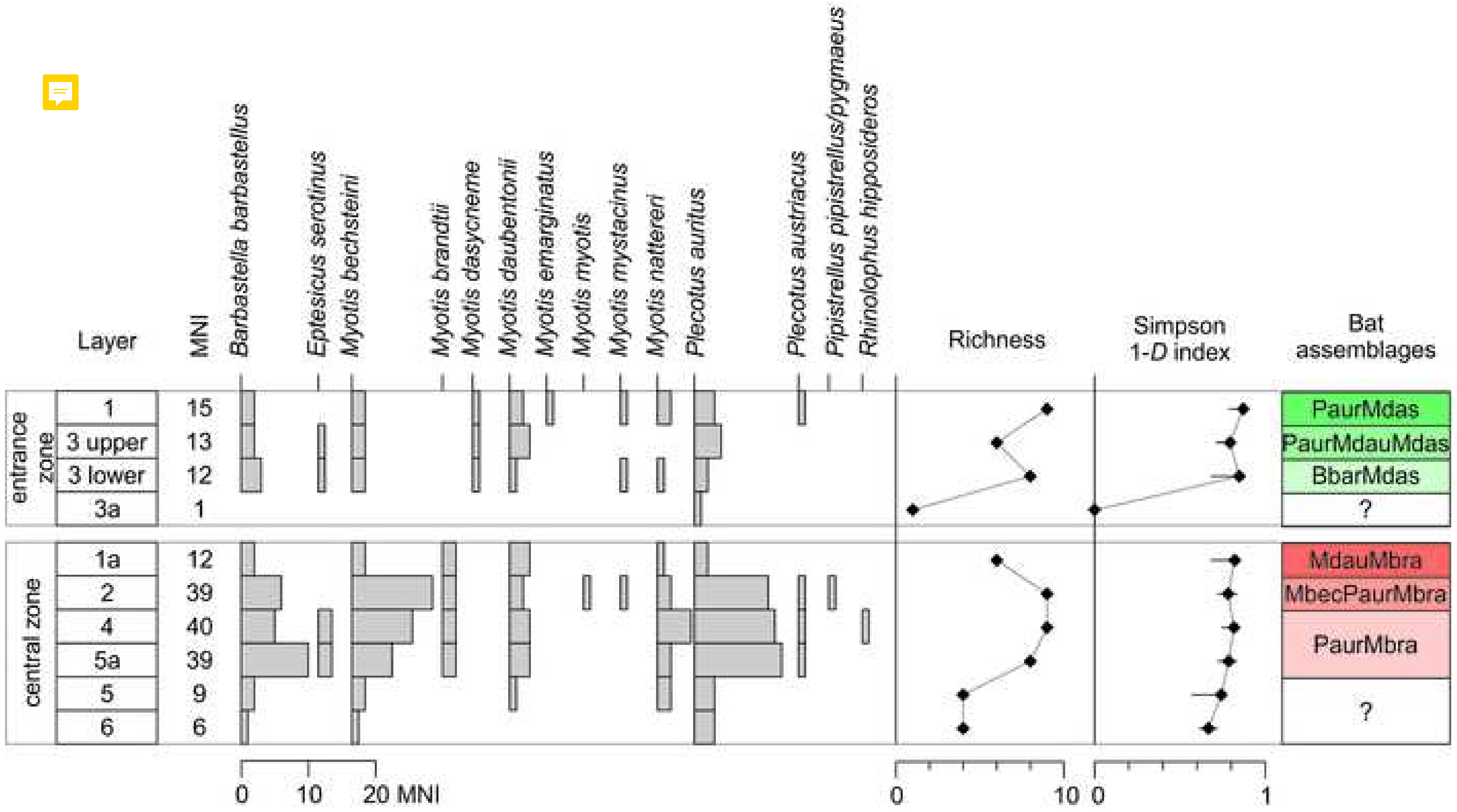

Figure 10

Click here to download Figure Figure 10.jpg

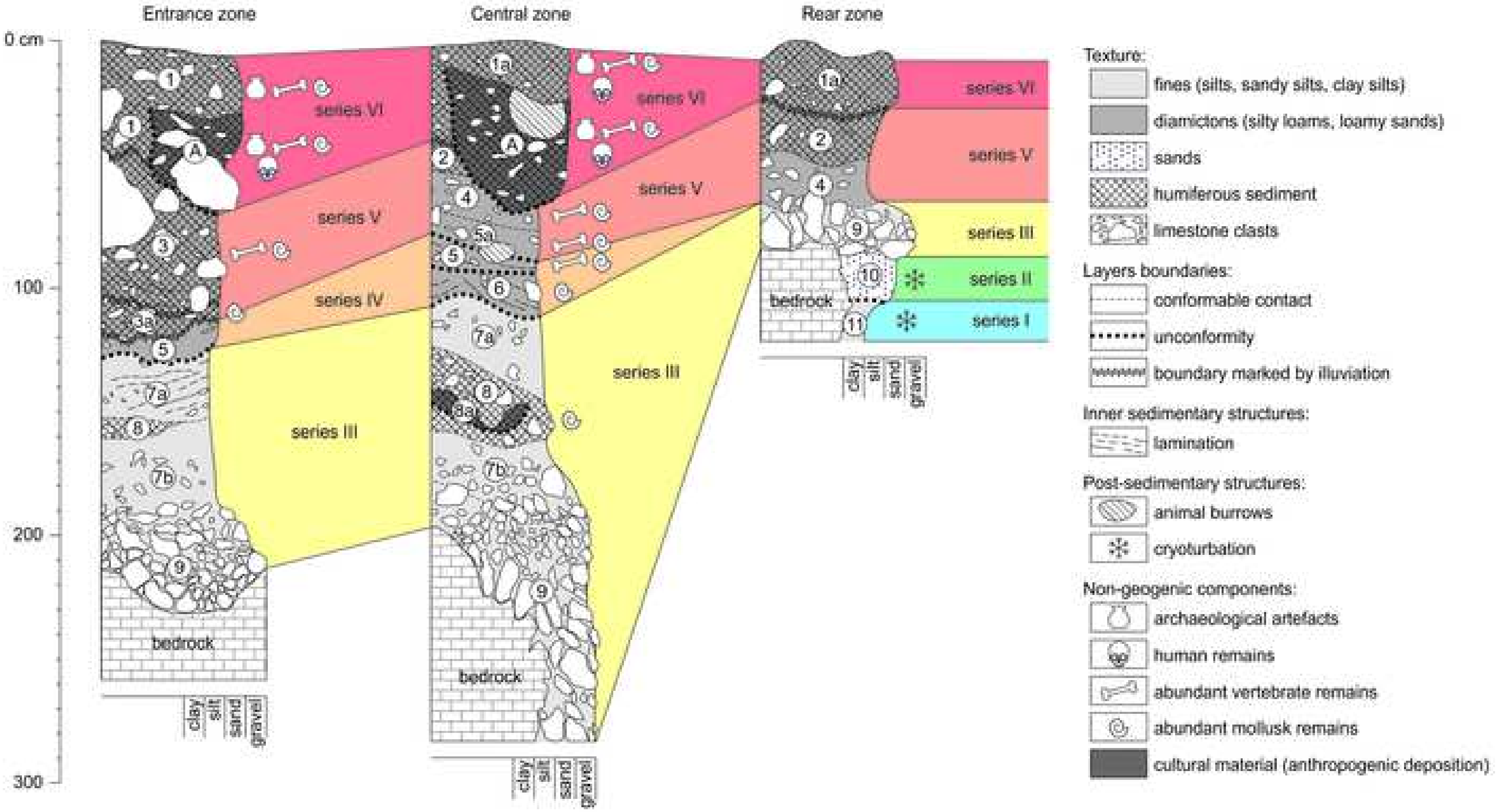

Figure 11

Click here to download Figure Figure 11.jpg

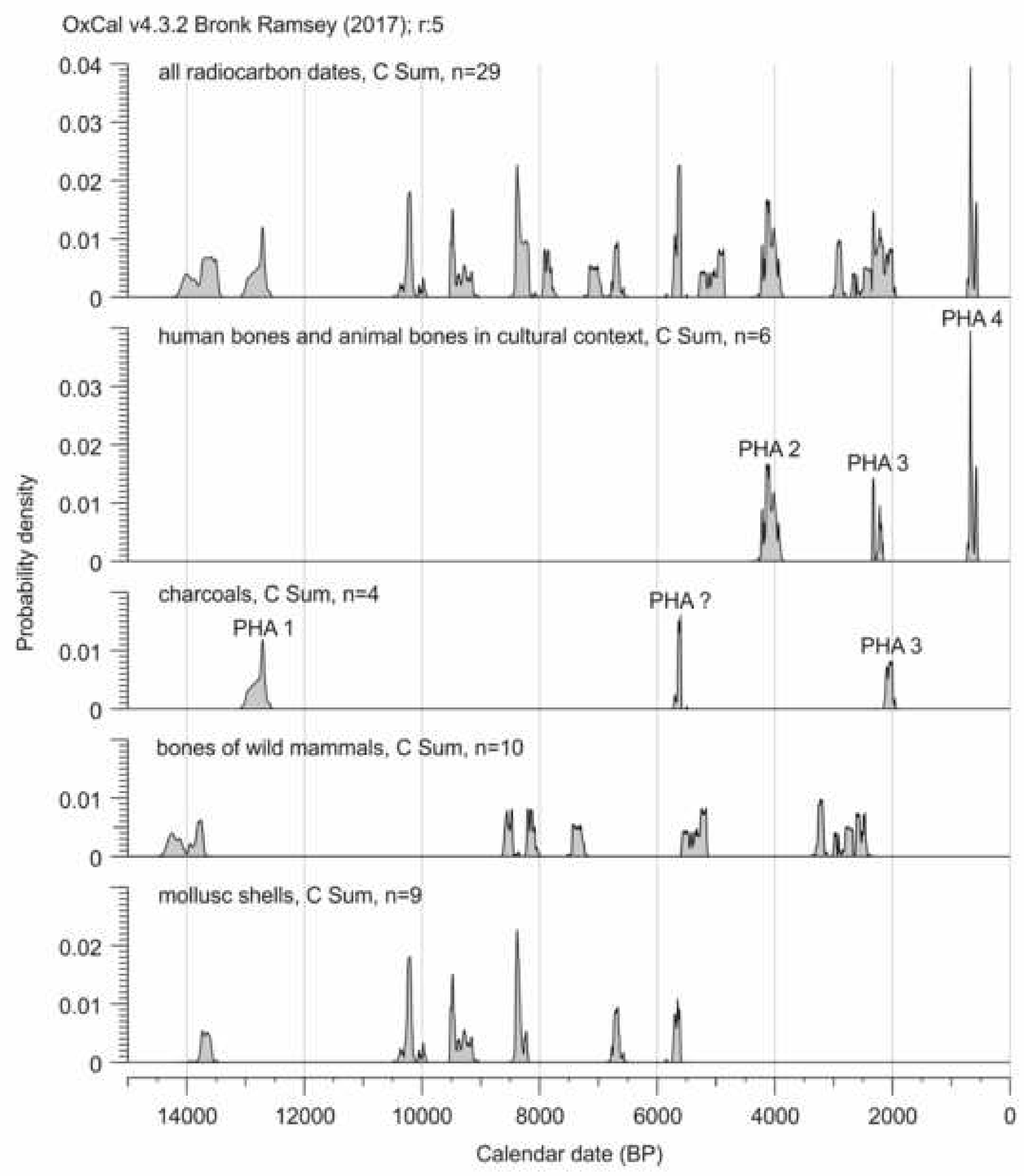

Figure 12

Click here to download Figure Figure 12.jpg

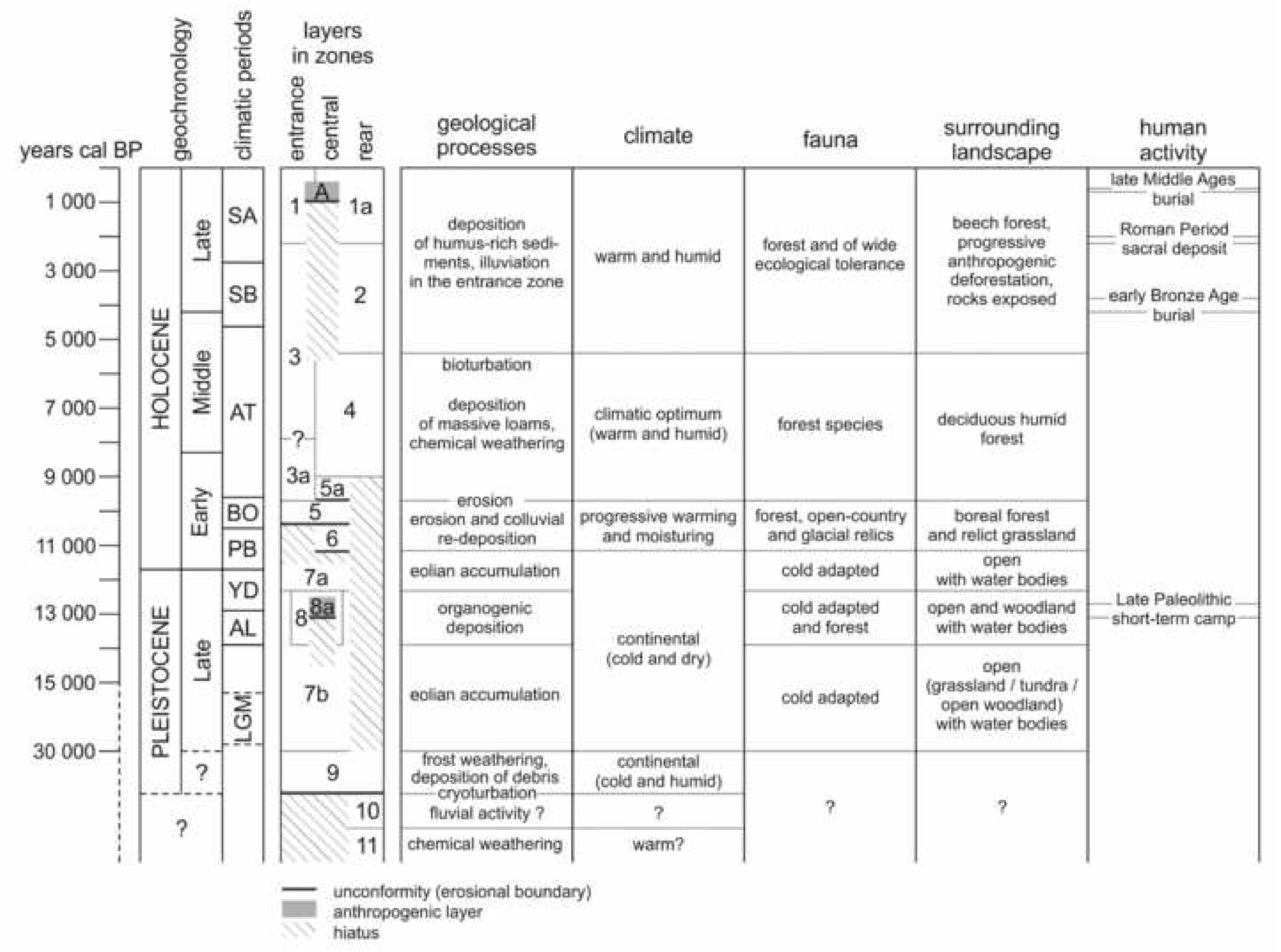

Click here to access/download
Supporting Information
Appendix S1 - lithology and geochemistry.xlsx

Click here to access/download
Supporting Information
Appendix S2 - Mollusks.xlsx

Click here to access/download
Supporting Information
Appendix S3 - Rodents.xlsx

Click here to access/download
Supporting Information
Appendix S4 - bats.xlsx
